# Genomics Portals: integrative web-platform for mining genomics data

**DOI:** 10.1186/1471-2164-11-27

**Published:** 2010-01-13

**Authors:** Kaustubh Shinde, Mukta Phatak, Freudenberg M Johannes, Jing Chen, Qian Li, Joshi K Vineet, Zhen Hu, Krishnendu Ghosh, Jaroslaw Meller, Mario Medvedovic

**Affiliations:** 1 Laboratory for Statistical Genomics and Systems Biology, Department of Environmental Health, University of Cincinnati College of Medicine, 3223 Eden Av. ML 56, Cincinnati OH 45267-0056, USA; 2 Department of Environmental Health, University of Cincinnati College of Medicine, 3223 Eden Av. ML 56, Cincinnati OH 45267-0056, USA

## Abstract

**Background:**

A large amount of experimental data generated by modern high-throughput technologies is available through various public repositories. Our knowledge about molecular interaction networks, functional biological pathways and transcriptional regulatory modules is rapidly expanding, and is being organized in lists of functionally related genes. Jointly, these two sources of information hold a tremendous potential for gaining new insights into functioning of living systems.

**Results:**

Genomics Portals platform integrates access to an extensive knowledge base and a large database of human, mouse, and rat genomics data with basic analytical visualization tools. It provides the context for analyzing and interpreting new experimental data and the tool for effective mining of a large number of publicly available genomics datasets stored in the back-end databases. The uniqueness of this platform lies in the volume and the diversity of genomics data that can be accessed and analyzed (gene expression, ChIP-chip, ChIP-seq, epigenomics, computationally predicted binding sites, etc), and the integration with an extensive knowledge base that can be used in such analysis.

**Conclusion:**

The integrated access to primary genomics data, functional knowledge and analytical tools makes Genomics Portals platform a unique tool for interpreting results of new genomics experiments and for mining the vast amount of data stored in the Genomics Portals backend databases. Genomics Portals can be accessed and used freely at http://GenomicsPortals.org.

## Background

A large amount of experimental data generated by modern high-throughput technologies is available through public repositories such as GEO [1] and ArrayExpress [2]. Our knowledge about molecular interaction networks and functional biological pathways is rapidly expanding and is being systematically organized into functionally related gene lists [3,4]. Jointly these two sources of information hold a tremendous potential for enhancing the interpretation of experimental results and gaining new insights into function of living systems. Mining such data has been a productive avenue in generating new hypothesis as well as validating experimental results [5]. Unfortunately, repositories currently housing much of the primary genomics data lack mechanisms for effective querying and analysis.

Inadequacies of the major data repositories to serve as access points to genomics data have resulted in numerous fragmented projects providing access to data from a single dataset [6,7], a set of thematically related datasets [8-10], or the results of genomics data analyses [11-15]. Except for the GeneChaser server [11], which provides access to results of differential expression analysis for all GEO DataSets, most of these resources are relatively small scale. Furthermore, they are generally focused on a single data type (mostly gene expression) and none of them facilitate the use of a functional knowledge base to construct query gene lists.

On the other end of the spectrum, several prominent efforts are directed towards constructing lists of functionally related gene lists [3,4], but they do not offer the capacity for querying genomics data based on these lists. The small exception is the capability of MSigDB server [4] to submit directly list of genes to the Gene Atlas server providing access to two microarray datasets [7].

The power of integrative analyses utilizing genomics data and functional knowledge has been demonstrated in the analysis of individual datasets [16] and systematic efforts to expand our understanding of gene functions [17-20]. In some cases, mining of new functional relationships predicted by integrative analysis of functional knowledge and genomics data is facilitated through predictive web servers [17]. Despite all these efforts, the integrated resources for accessing and analysis of both functional knowledge and the primary genomics data on a large scale are still lacking. Genomics Portals platform was designed to fill this gap.

The access to gene expression regulatory data such ChIP-chip and ChIP-seq transcription factor binding and epigenomics data is even more difficult and fewer resources are available. Most of the datasets are still deposited to the main genomics repositories. However, the only meaningful way to access this data is through UCSC and ENSEMBL Genome Browsers [21,22]. Both of these browsers are genomic feature - centric and do not provide meaningful analysis options and graphical displays for multiple gene promoters at the same time. On the other hand, presenting such data using heatmaps of many genes at a time has been commonly used in publications and is an effective way of exposing patterns in such data [23]. In the spirit of "group of genes queries" used throughout Genomics Portals, we facilitate the analysis and graphical presentation of this data in the form of heatmaps for a fixed window around the transcription start site and the whole list of genes at a time. We are unaware of any server other than Genomics Portals that offers similar functionality for accessing and analyzing this kind of data.

Genomics Portals platform was designed around the three conceptual problems faced daily by biomedical scientists:

1. **Characterizing experimentally derived gene lists in the context of relevant publicly accessible genomics data (>82,000 genome scale data vectors; more than 1.8 billion data points)**. By simply pasting the experimentally derived gene list (e.g. differentially expressed genes, co-expressed genes, transcription factor regulated genes, epigenetically modified genes, etc) into the query box and then selecting the relevant datasets, one is able to download the primary data, perform basic analysis and generate publication-quality graphics depicting the expression patterns of the genes queried. The whole process can take less than one minute.

2. **Functional analysis of newly generated data**. By depositing newly generated data into Genomics Portals databases, one can leverage extensive knowledge base (>20,000 gene lists specific to biological pathways, diseases, transcriptional factor regulatory domains, etc), or browse our collection of analytical results to construct meaningful gene lists for querying their own data.

3. **Integrative mining of public genomics data**. Researchers can simply use the knowledge base and their own imagination to construct query gene lists and select genomics datasets to mine.

**Figure 1:**
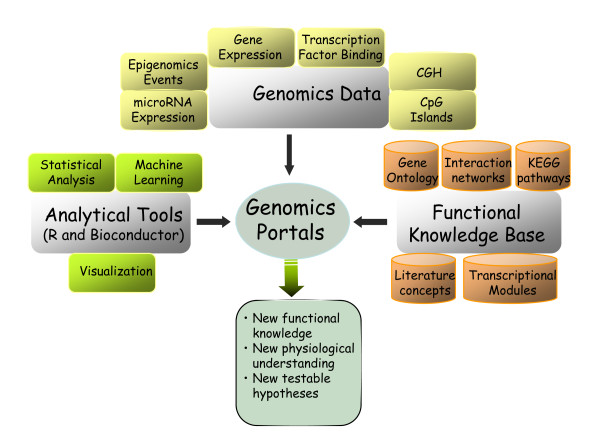
**Conceptual structure of Genomics Portals**. Diverse types of whole genome datasets are integrated with the functional knowledge base and basic statistical and machine learning procedures into a comprehensive data mining environment.

**Figure 2:**
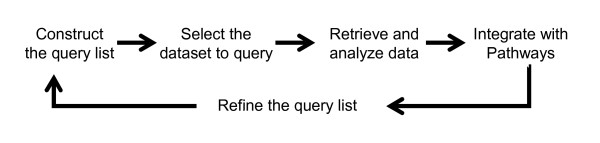
**Typical analysis flow**. A typical analysis (Figure 2) starts by constructing a list of genes by either using the predefined lists or pasting a gene list of interest, querying one of the databases with genome-scale data, and producing analysis summaries. Based on the analysis results, one can further refine their gene query list and repeat the procedure on a different dataset.

The conceptual structure of Genomics Portals is depicted in Figure 1. The Figure emphasizes the integrative nature of the platform. Diverse types of whole genome datasets are integrated with the functional knowledgebase and basic statistical and machine learning procedures into a comprehensive data mining environment. A typical analysis (Figure 2) starts by **constructing a list of genes **by either using the predefined lists, or pasting a gene list of interest; **querying one of the databases **with genome-scale data; and **producing analysis summaries**. Based on the analysis results, one can further refine their gene query list and repeat the procedure on a different dataset.

## Implementation

### Genomics Portals knowledge base and gene list construction

Genomics Portals knowledge base consists of >20,000 lists of functionally related genes, Entrez GeneRIFs, and BioGRID gene-gene interaction data. One can construct a query gene list by pasting their own list, by manipulating the predefined gene lists, by an open text search of GeneRIFs, and by searching for interaction partners of a gene or a group of genes. One can also elicit a gene list by browsing pre-computed clustering results for different datasets.

Numerous efforts are underway to systematically collect and organize the functional information about genes. Our first goal is to assemble lists of functionally related genes from such collections. Following are sets of gene lists we gathered using publicly available resources: Gene Ontologies [24], KEGG pathways [25], Mouse Phenotypes [26], L2L lists of published differentially expressed genes [27], and miRBase predicted microRNA targets [28]. Additionally, we created lists of computationally predicted transcription factor targets, disease related gene lists based on text-processing GeneRIFs, and a limited set of custom created gene lists taken from the literature that were not found in the L2L database.

For a most of gene expression datasets we also performed unsupervised cluster analysis followed by in-depth functional annotation of the clustering structure. The cluster analysis was performed using the Bayesian model-based procedures [29,30] as well as simple hierarchical clustering. The functional annotation of the clustering structures was performed using the CLEAN framework [31]. The integrative browsing of the data and functional annotations is facilitated through the Functional TreeView (FTreeView) application which is a Java web-start based clustering browser [31] developed based on the open source Java TreeView browser [32]. Using FTreeView, one can identify clusters of genes based on their data profile and correlation with specific functional categories and use such gene lists to query and analyze genomics data in other datasets.

### Genomics Portals genomics data

The vast majority of the genomics data deposited in our databases is public data downloaded from the major repositories (GEO, ArrayExpress, and UCSC Genome Browser), or produced by the computational analyses of genomics data (e.g. computationally predicted transcription factor binding sites). A small portion of the database is private data belonging to our collaborators which is accessible after a log-in. No registration or login is required for accessing the public data. In general, "genomics data" refers to genome-scale vector of measurements produced by various experimental assays (expression microarrays, CGH microarrays, ChIP-chip and ChIP-seq, etc.) or computationally constructed scores (CpG islands, transcription factor binding scores and microRNA target scores). Genomics datasets are organized thematically into different data portals. Different portals can contain datasets related to different diseases (e.g. Breast Cancer and Prostate Cancer), specific types of genomics data (e.g. Epigenomics and Transcription Factors), or different biological processes (e.g. Development). The same dataset can be assigned to different portals.

### Gene expression data

Gene expression data in our databases consists of the complete collection of human, mouse and rat GEO DataSets (>1,500), and close to 100 manually curated GEO series of particular interest. The majority of the manually curated datasets are not available as GDS DataSets. For some of the manually curated datasets we have re-processed the raw outputs (e.g. CEL and GPR files).

### ChIP-chip and ChIP-seq data

Most of these datasets have been manually curated and re-processed. The data is summarized as the average measurement intensities within non-overlapping 50 bp windows for the -4 kb to +1 kb regulatory region around each RefSeq sequence for the given genome build. This representation provides for a straightforward manipulation and graphical representation of such data for gene lists. The definition of "measurement intensities" depends on the primary data that was downloaded from the repository and it can range from typical log2-scaled fluorescence intensities for hybridization-based experiments to sequence read counts and various approaches to identifying and quantifying "peaks" for high-throughput sequencing technologies.

### Computationally constructed scores

Most of this type of data is derived from computational assessments of DNA features within the genomic regulatory regions such the existence of transcription factor (TF) binding motifs and CpG islands. Computationally predicted transcription factor binding scores are provided as whole gene (i.e. Refseq) scores that assess the overall likelihood of a transcription factor binding within the gene's promoter and high resolution datasets that provide locations of putative TF binding sites at the same resolution used for ChIP-chip and ChIP-seq data. All scores were calculated using in-house developed scoring algorithms and TRANSFAC transcription factor binding motif definitions [33].

### Genomics Portals analysis tools

In designing Genomics Portals we sought to strike a balance between the key limiting factors such as the complexity of query interfaces and the computational complexity of the analyses performed on the data, and the usefulness of the results produced. The portal is designed so that a basic output of the genomics data queries can be obtained in less than one minute with only a few clicks of the mouse. This includes query of our knowledge base or pasting one's own gene list, selecting the genomics data to query, and retrieving the data in the form of a spreadsheet or an R ExpressionSet. At this point, the user can either use their own data analytical tools or perform basic manipulation and analysis of the retrieved data within the portal. Subsequent analyses are performed using the retrieved data and the simple interface that deploys R and Bioconductor procedures using the *Rserver *infrastructure. The basic manipulations consist of sub-setting the data, selecting grouping variables for the samples in the datasets, identifying differentially expressed genes, cluster analysis, assessing enrichment of differentially expressed genes within the query list, etc. The results of such analyses are provided as static annotated heatmaps, or can be browsed using our FTreeView browser. Analyses of results can also be incorporated within the images of KEGG pathways.

### Genomics Portals computational infrastructure

**Figure 3:**
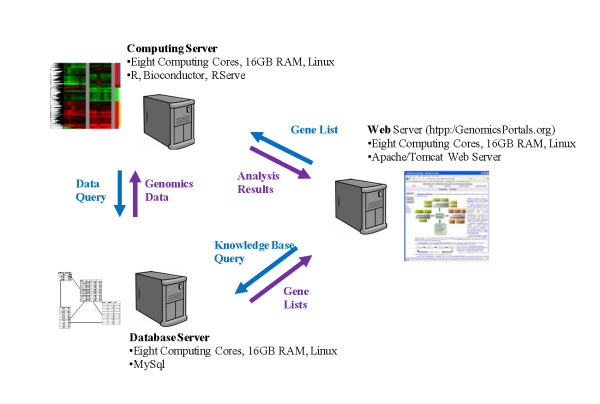
**Hardware infrastructure**. The hardware infrastructure mirrors three basic computational modules and consists of the database server containing all MySQL databases, the web-server, and the computational server that runs RServer and is used for performing computational tasks. The modularity of the infrastructure ensures the responsiveness of the web server even under heavy computational load and effectively leverages computational resources of three different 8-core servers.

The basic computational infrastructure behind Genomics Portals consist of Java-based web interfaces and data viewers, *MySQL *databases for organizing knowledge base and genomics data, and R scripts for performing analysis of retrieved data using the RServer infrastructure to connect Java with R. The relational databases storing genomics data are loosely based on the *MySQL *version of ArrayExpress, MaxD http://www.bioinf.man.ac.uk. Query gene lists are constructed and genomics datasets selected using Java-based interfaces. Java programs then call appropriate R scripts that query genomics databases (using *RMySQL*) and perform the analysis using various Bioconductor packages. The hardware infrastructure mirrors these three basic computational modules (Figure 3) and consists of the database server containing all *MySQL *databases, the web-server, and the computational server that runs *RServer *and is used for performing computational tasks. The modularity of the infrastructure ensures the responsiveness of the web server even under heavy computational load and effectively leverages computational resources of three different 8-core servers.

### Maintenance and Updates

Processing and uploading of all data in back-end databases are performed by executing standardized R scripts utilizing *RMySQL *package. All scripts performing any kind of operation on the back-end databases are documented and archived. In principle, we are able to re-create all databases from raw data by re-running archived R scripts. All updates to the data housed in the back-end databases are semi-automated, meaning that standardized R scripts performing updates are manually executed. Three aspects of the portal are periodically updated:

• The gene annotation table which is constructed by combining *gene_info *and *homologene.data *tables downloaded from respective NCBI ftp sites.

• Gene lists constructed from public annotation efforts (GO, KEGG, L2L and miRBase) are periodically updated by executing appropriate R scripts. For GO and KEGG updates we utilize most recent Bioconductor data objects. For L2L and miRBase lists, we download the current version from the primary web sites.

• For gene lists constructed by analyzing public data (Disease gene lists and Mouse phenotype gene lists), the process is similar except it proceeds in two steps. The current version of primary data (Gene RIFs and Mammalian phenotype ontologies) is downloaded and re-analyzed using existing R scripts to construct the gene list which are then uploaded to the back end database.

Processed primary genomics data is continually added, but there are no planned updates of the data that has already been uploaded. Each curated dataset is associated with a standardized R script that was used to process the raw data and upload the dataset to the back-end database. When an error is detected in any of the datasets, we generally remove the dataset, correct the error in the script used to upload the dataset and re-upload the dataset. This way, each manipulation of the data is documented by the R program that performed it. The download and processing of curated GEO data sets (GDS) was performed in batch using again specifically developed R programs, and new datasets will be periodically added as they become available using the same scripts.

## Results

### Case Study: Characterizing experimentally identified proliferation signature

We demonstrate the utility of the Genomics Portals through a case study investigating a proliferation gene expression signature in rat mammary epithelium induced by different fatty acid diets [34]. That study established the increased proliferation of mammary epithelium as a consequence of several different dietary regiments in virgin female Spraque-Dawley rats. The study also identified a set of 85 genes whose expression levels were correlated with the increased proliferation. We used Genomics Portals to study the functional importance of these genes in five different biological processes examined in four gene expression datasets (Miller, Fournier, Herschkowitz, and Moggs) [35-38]. All analyses shown here were performed using the Genomics Portals web-interface. The step-by-step instructions for reproducing these results are described in Genomics Portals User Manual which is provided in additional file 1 as well as in the online help.

**Figure 4:**
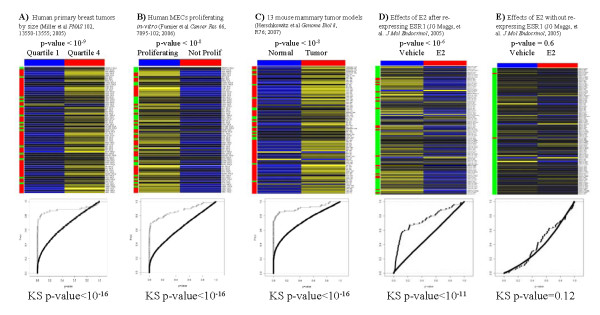
**Proliferation signature in four transcriptional datasets**. Genomics Portals were used to study the functional importance of 85 genes constituting in the rat mammary cell proliferation signature in five different biological processes examined in 4 gene expression datasets. **A) **The comparison of between the largest (top quartile) and smallest (bottom quartile) tumors with the assumption that large tumors are "more proliferative" than small tumors in the Miller dataset. **B) **The comparison between the proliferating and growth-arrested cell lines in the Fournier expression dataset. **C) **The comparison between all 13 different murine breast cancer models and normal controls in the Herschkowitz expression dataset. **D) **The transcriptional effects of estrogen on ER- breast cancer cell line after re-expression ESR1 in the Moggs expression dataset. **E) **The transcriptional effects of estrogen on ER- breast cancer cell line without re-expression ESR1 in the Moggs expression dataset.

All four gene expression datasets used in the analysis investigate proliferation-related biological processes. For each of the datasets, we assessed the enrichment of differentially expressed genes in specific comparisons among the proliferation genes identified in the rat dietary study (Figure 4). For each comparison of interest, Genomics Portals scripts calculated Empirical Bayes p-values using the *limma *package [39] for the selected genes and a randomly selected list of probes of the same length. The enrichment of the statistically significant genes in the query list was then assessed using logistic regression (LRpath) [40]. The query list naturally consisted of rat gene identifiers which were automatically translated to appropriate human and mouse homologs while executing queries.

The Miller dataset [35] is a well-annotated gene expression dataset profiling 251 primary human breast tumors. This dataset was re-processed and curated before deposited into the back-end databases under the id "GSE3494Entrez". The comparison of interest in this case (Figure 4A) was between the largest (top quartile) and smallest (bottom quartile) tumors with the assumption that large tumors are "more proliferative" than small tumors. Indeed the genes in the query list were up-regulated in large tumors and enriched for differentially expressed gene (LRpath p-value < 10^-9^).

The Fournier dataset [36] (Genomics Portals id "EGEOD8096Entrez") profiles gene expression of growth-arrested human mammary epithelium in-vitro. The comparison made was between the proliferating and growth-arrested cell lines (Figure 4B). Again, query genes were generally down-regulated in growth-arrested mammary acini and were enriched for differentially expressed genes (LRpath p-value < 10^-8^).

The Herschkowitz dataset [37] (Genomics Portals id "GSE3165gpl891") compares gene expression profiles of 13 different murine breast cancer models. The comparison made in our analysis was between all tumor tissues and normal controls (Figure 4C). Query genes were generally up-regulated in tumor tissues indicating, as expected, higher proliferation when compared to normal mammary tissue (LRpath p-value < 10^-8^).

The Moggs dataset [38] (Genomics Portals id "gdsGDS1326") examines transcriptional effects of estrogen on ER- breast cancer cell line after re-expression ESR1. While estrogen exposure generally increases the proliferation of ER+ breast cancer cells, in the ER- cell line with re-expression of ESR1 estrogen exposure reduces the proliferation. In our analysis we compared expression levels of our gene list before and after estrogen exposure in for both ER- cell line (MDA-MB-231) with and without re-expression of ESR1. In concordance with the observed phenotype, for the cell line with re-expressed ESR1 genes in our list are repressed after estrogen treatment (Figure 4D) in the statistically significant fashion (LRpath p-value < 10^-6^). For the same cell line without re-expressing ESR1, there was no discernible effect on expression of genes in our list (Figure 4E) after estrogen treatment (LRpath p-value = 0.6). Interestingly, the majority of genes in the re-expression experiments (Figure 4D) did not individually show statistically significant differential expression (at p-value < 0.05 level). However, jointly, the distribution of their p-values was strongly enriched for small p-values in comparison to randomly selected list of genes.

We established the universality of the proliferation signature identified in the rat dietary studies across four very different biological systems. Using the Genomics Portals interface, the entire process of querying and generating results in Figure 4 can be completed in less than 10 minutes. The strategy of assessing the statistical significance of enrichment by differentially expressed genes by comparing the query list p-values to the randomly selected probes is implemented to reduce the computational burden that would be imposed by retrieving data and calculating p-values for all probes. We assessed the validity of this strategy for the four datasets at hand by examining off-line the empirical cumulative distribution functions of p-values for genes in the query list (open circles) and genes probes not associated with genes in the query list (solid line) (Figure 4). For all four situations yielding statistically significant LRpath tests, the query list was obviously enriched for differentially expressed genes, which was also confirmed by the Kolomogorov-Smirnov test for differences between the two distributions. For the non-statistically significant comparison (Figure 4E), there was no difference between the empirical distributions functions for all probes (Figure 4E).

**Figure 5:**
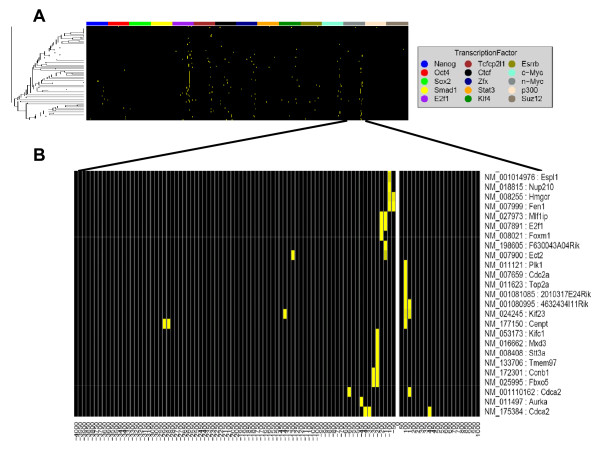
**Proliferation signature in transcription factor binding ChIP-seq data**. Genomics Portals were used to examine the promoter binding events in the ChIP-seq dataset assessing DNA binding of 15 different transcription factors, including E2F1, in mouse embryonic stem cells. **A) **Binding patterns for all 15 transcription factors in the promoter regions (-4 kb to +1 kb around TSS) of the 85 genes constituting the rat mammary epithelium proliferation signature. **B) **n-Myc binding patterns in the promoters of a subset of the genes constituting the proliferation signature. This higher-resolution figure was generated by filtering-out other 14 transcription factors.

In addition to using gene expression data, we further characterized our proliferation signature using ChIP-seq data for E2F1 transcription factor (TF) [41] and by comparing histone modification marks between differentiated mouse embryonic fibroblasts (MEF) and a stem-cell "like" cell line [42]. In the original paper, an extended set of genes identified through cluster analysis was linked to regulatory domain of E2F transcription factors by examining the overlap with E2F targets established in ChIP-chip [43] and global expression profiling [44] experiments, and computationally predicted E2F targets. Here we used Genomics Portals to examine the newer ChIP-seq dataset assessing DNA binding of 15 different transcription factors, including E2F1, in mouse embryonic stem cells (Figure 5). In addition to most of the genes having a ChIP-seq peak for E2F1 within the regulatory region examined (-4 kb to +1 kb around TSS), there were several other transcription factor that seemed to have unusually many peaks for these genes. To test this hypothesis we again used the comparison to a "random" sample by LRpath. Instead of the p-values, in this situation Genomics Portals by default uses the maximum "peak intensity" calculated for each gene across its whole regulatory region. Such statistical analysis confirmed that in addition to E2F1 (p-value < 10^-14^), n-Myc (p-value < 10^-7^), Tcfp2l1 (p-value < .001), c-Myc (p-value < .01), and Klf4 (p-value < 0.01) all show signs of increased binding to regulatory regions of these genes.

**Figure 6:**
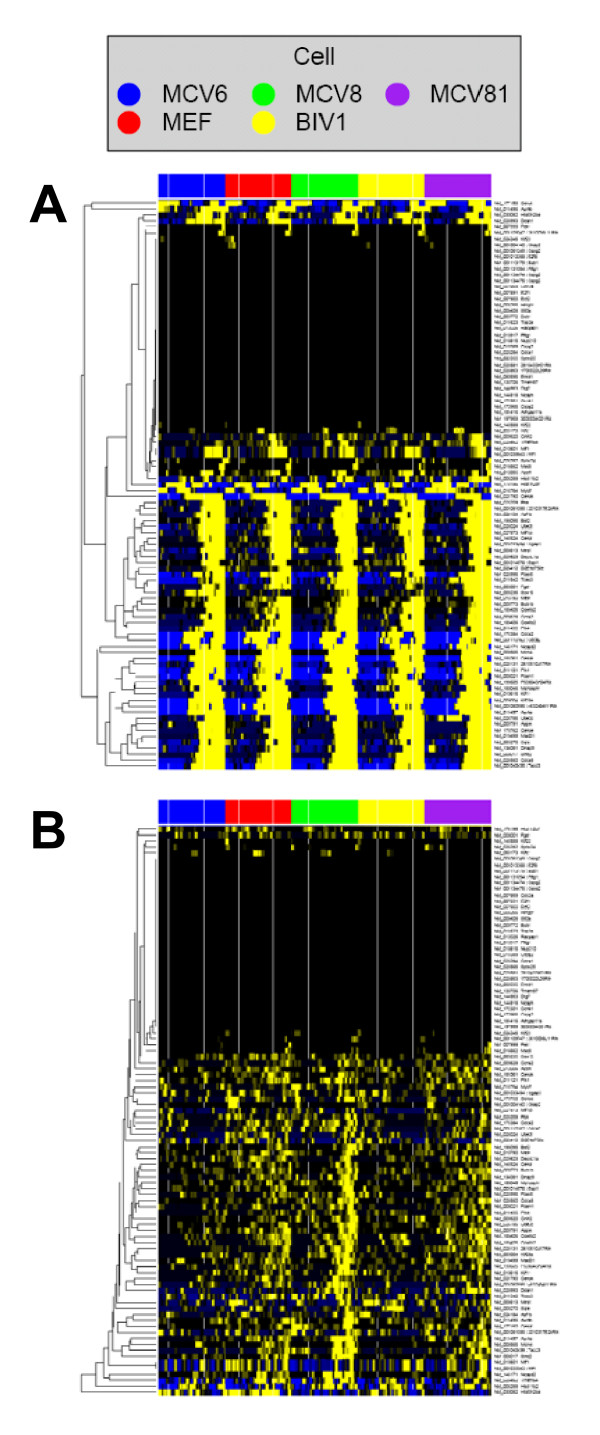
**Proliferation signature in epigenomics ChIP-seq data**. Genomics Portals were used to examine patterns of two epigenomics histone marks, H3k4me3 (**A**) and H3k27me3 (**B**) across five human cell line at different "differentiation" stages.

A similar analysis of two epigenomics histone marks, H3k4me3 (Figure 6A) and H3k27me3 (Figure 6B) across five human cell line at different "differentiation" stages [42] indicate that there is a subset of genes in our proliferation signature with strong tri-methylation of histone 3's lysine 4 across all 5 cell lines. On the other hand, tri-methylation of histone 3's lysine 27, in addition to differences between genes, also shows differences between different cell lines. Further examinations of functional relevance of these observations are beyond the scope of this paper.

## Discussion

By constructing Genomics Portals we sought to provide meaningful access to diverse genomics datasets currently residing in major public repositories for biomedical researchers without much technical bioinformatics expertise. There is a myriad of bioinformatics solutions that facilitate download and analysis of such datasets. For example, to process and populate our database we used R [45] and Bioconductor packages [46]. However, to acquire data and perform appropriate analyses using these tools requires a certain level of technical bioinformatics expertise. Genomics Portals allows researchers without such expertise to perform meaningful analysis of these datasets.

The general strategy of focusing on lists of functionally related genes as the basic unit for querying and analyzing genomics data is in-line with current thinking that specific functions of a living system are conferred by a coordinated action of a specific set of genes. In this paradigm, a single gene analysis is superseded by the joint analysis of all genes that are functionally related under certain conditions. The utility of such approaches has been demonstrated in numerous studies and confirmed in the case study we presented in this manuscript. Of course, a single gene can be treated as the list of size one and Genomics Portals allows queries and analysis based on the single gene as well. The second guiding idea in constructing the platform is the need to integrate functional data (gene expression), regulatory events data (epigenomics, TF binding) and the knowledge base (lists of functionally related genes).

Our decision to use R as the analytical engine within Genomics Portals provides us with virtually limitless possibilities for the analysis of data acquired in a given query. In the current implementation we sought to strike a balance between the key limiting factors such as the complexity of query interfaces and the computational complexity of the analyses performed on the data, and the usefulness of the results produced. This resulted in a highly streamlined interface with relatively few analytical options. Users who wish to perform additional off-line analyses can do so by downloading the data retrieved in the query. In future implementations we plan to "expose" more of the Bioconductor functionality through an additional "Advanced Analysis" web-interface while still keeping the current simple interface as the main option.

Other natural extensions of current infrastructure will be allowing for simultaneous queries of multiple datasets and addition of data from other organisms. The implementation of simultaneous queries of multiple datasets will be straightforward. However, query times and computational resources required to perform such queries will be additive in the number of datasets queried. This option will be included once we ascertain that our computational infrastructure is sufficiently robust to provide the current level of interactivity at such increased level of functionality. Expanding the data coverage to any other organism covered by Entrez Gene identifiers will also be rather straightforward. Since genomics data for different species always reside in different databases, adding such data will almost certainly not affect the performance of the portal.

The case study presented here is demonstrating the usage of only a small portion of the platform. Comprehensive online documentation is provided for complete description of the data and analytical options available through Genomics Portals.

## Conclusions

Genomics Portals represents a powerful new tool for gaining knowledge from results of new genomics experiments as well as for mining a large collection of primary genome-scale datasets.

## Availability and requirements

Project name: Genomics Portals

Project home page: http://GenomicsPortals.org

Operating system: platform independent

Programming language: Java, MySQL, R

Other requirements: None

License: The tool is available online free of charge

Any restrictions to use by non-academics: None

## Authors' contributions

KS developed the database infrastructure, tools for populating databases and web-interfaces for querying databases, MP participated in the overall design, developed the protocols and wrote R scripts for performing the analyses and producing graphical displays, MM conceived the portal, developed initial scripts for processing and uploading data and guided the development of all aspects of the portal, JF processed and analyzed a significant number of public datasets, JC developed protocols for processing and uploading, and processed most of the ChIP-chip and ChIP-seq data, QL processed and analyzed a significant portion of curated datasets, ZH computed the predicted transcription factor binding sites, VJ developed and integrated the FTreeView browser, KG assisted with scoring computationally the likelihood of transcription factors binding, JM provided important feedback through incorporating the use of the portal in several graduate courses he is teaching. All authors read and approved the final manuscript

## Supplementary Material

Additional file 1 **Genomics Portals User Manual**. The online help file containing screenshots and step-by-step instructions on how to use Genomics Portals. Click here for file
