# Supplementary material for: Genomics Portals: integrative web-platform for mining genomics data
Source: BMC Genomics. 2010 Jan 13;11:27. doi: 10.1186/1471-2164-11-27 (PMC2824719; doi:10.1186/1471-2164-11-27)
Supplement: Additional file 1 — Genomics Portals User Manual. The online help file containing screenshots and step-by-step instructions on how to use Genomics Portals. [file 1471-2164-11-27-S1.PDF]

Contents

1 Introduction **2**

2 Start by constructing a gene list **2**
- 2.1 Using predefined gene list(s) 2
- 2.2 Search for genes of interest using entrez id, symbol or description. 5
- 2.3 Paste a list of genes in the box provided. 5
- 2.4 Find predefined gene list(s) for your choice of genes. 6
- 2.5 Find genes with a phrase in their RIFs. 7
- 2.6 Find biogrid gene pairs for your gene(s). 8

3 Start by selecting an experiment **8**
- 3.1 Search for an experiment 9
- 3.2 Query experiment 10
- 3.3 Cluster experiment 10

4 Miscellaneous modules **11**
- 4.1 Filter samples and select sample grouping for analysis 11

5 Interpreting Results **12**
- 5.1 Interactive Treeview Browsing 12
- 5.2 Static heatmaps 13
  - 5.2.1 Statistical Analysis 14
- 5.3 Gene List Statistics 14
  - 5.3.1 Predictive Ability Pvalue (LR) 14
- 5.4 Kegg Pathways for submitted genes 15

6 Case Study: Characterizing experimentally identified proliferation signature **15**
- 6.1 Gene Expression data 16
  - 6.1.1 Select a dataset from the portal 16
  - 6.1.2 Paste a query gene list 16
  - 6.1.3 Select Sample Grouping 16
  - 6.1.4 Results 17
- 6.2 ChIP-seq data for different transcription factors 17
  - 6.2.1 Select a data set from the portal 18
  - 6.2.2 Paste a query gene list 18
  - 6.2.3 Select Sample Grouping 18
  - 6.2.4 Results 18
- 6.3 Tri-methylation of histone across 5 human cell lines 19
  - 6.3.1 Select a data set from the portal 19
  - 6.3.2 Paste a query gene list 20
  - 6.3.3 Select Sample Grouping 20
  - 6.3.4 Results 20

1 Introduction

GenomicsPortals is a web-based integrative computational platform for the analysis and mining of genomics data. We aim to integrate the primary genomics data, functional knowledge base and analytical tools within a single framework.

Genomics datasets are organized thematically into different portals. Different portals can contain datasets related to different diseases (eg Breast Cancer and Prostate Cancer), specific types of genomics data (eg Epigenomics and Transcription Factors), or different biological processes (eg Development). The same dataset can be assigned to different portals.

A typical analysis starts by constructing a list of genes by either using the predefined lists of pasting a gene list of interest, querying one of the databases with genome-scale data and producing analysis summaries. One can also start by searching for dataset of interest, and then constructing the query gene lists. In this case, one can also construct gene lists by browsing pre-computed clustering results.

We would like to note that we have designed the layout with the font size of 16 as a reference. If required, this default font size can be changed in the browser to increase the readability. In certain cases, simply "zoming in" will also make the text easier to read without pictures going out of focus.

2 Start by constructing a gene list

There are many ways to construct a gene list.

- Use a predefined gene list.
- Search for genes of interest using entrez id, symbol or description.
- Paste a list of genes in the box provided.
- Find predefined gene list(s) for your choice of genes.
- Find genes with a phrase in their RIFs.
- Find biogrid gene pairs for your gene(s).

The above list depicts various starting points to generate a list of genes of your interest. Rest of the work-flow is quite similar no matter how one selects a gene list.

2.1 Using predefined gene list(s)

Figure1 shows the interface to select a predefined gene list. Clicking on "Gene List" tab in the left menu would get this page. The lists are organized in different categories and we are constantly adding new lists and categories. Let's say we are interested in gene lists in category "KEGG" with keywords "cell cycle". Click

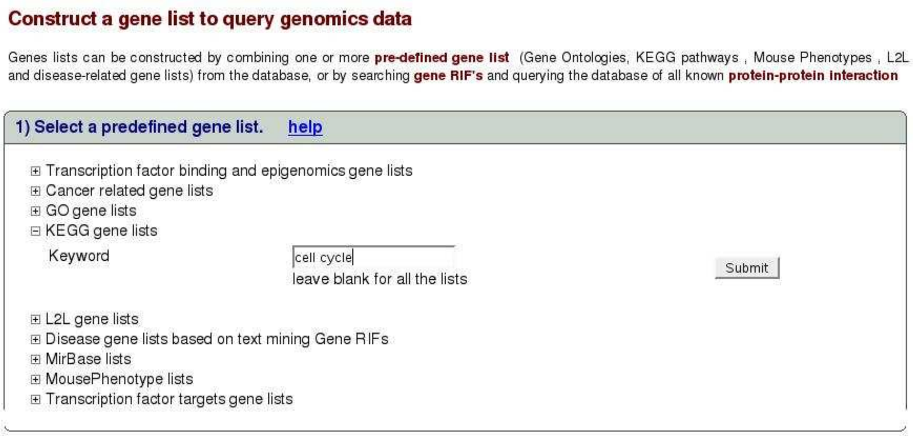

Figure 1: Search a predefined gene list

on the link "KEGG gene list" to expand the search box as shown below. Type cell cycle in the text box and click submit.

This takes us to the following screen (figure2). Here we see a list of gene list returned for the keywords. Select one or multiple lists using the check boxes and click submit.

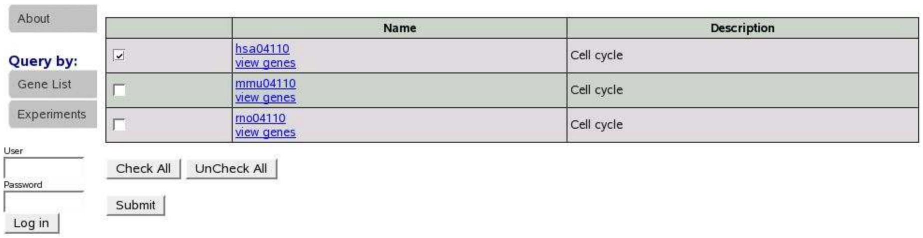

Figure 2: Select gene list

Now we see summary for the submitted lists. Note that below the summary table, we are asked to select union or intersection of the gene lists selected. In this case, because we submitted only one gene list, both union and intersection are identical. Let's select union and proceed to select an experiment for analysis.

Experiments are organized into different portals. If we know the portal the experiment of our interest belongs to, we can click on the portal name to list all the experiments in that portal. Since this is not the case most times, a search function is provided to look for experiments of our interest. In the "Filter experiments" box shown below, select organism, sample type, portal of your interest and type in keyword. Keyword could be left blank.

Let's click on portal name "Breastcancer" to proceed with our example.

Figure5 is a part of the screen showing experiments in the portal Breast-

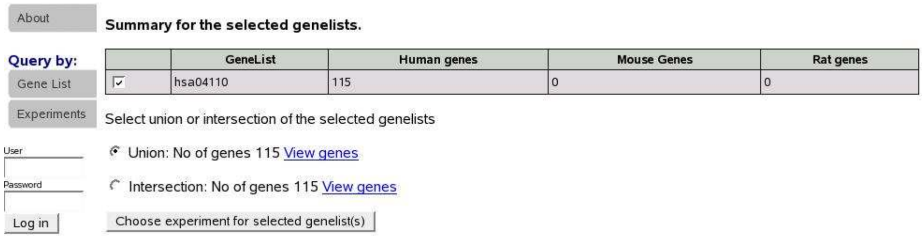

Figure 3: Summary of gene list

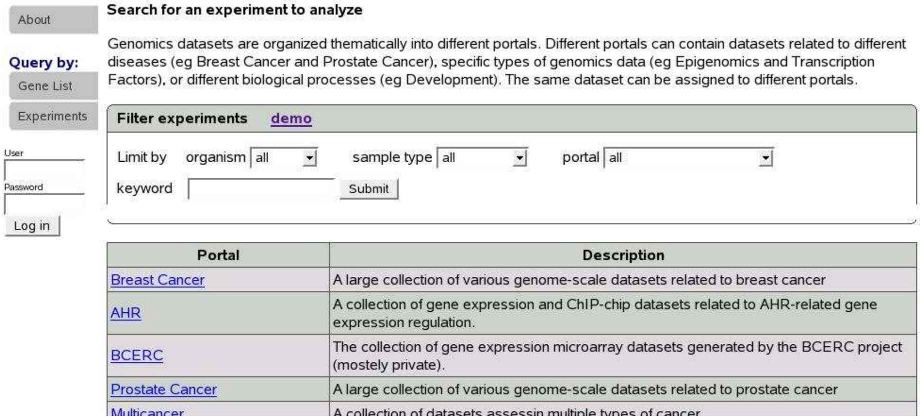

Figure 4: Select or search for experiments

cancer. If we had used the search function to look for an experiment instead, a similar screen would be shown. Below you would see a list of experiments. Let's select first experiment (GSE10797) and scroll down and click submit.

At this point, data is retrieved from database for the selected experiment and gene list(s) as shown in figure6.

One could download the data for his/her own analysis either as a tab delimited file or an eset for analysis in R. In this example, we have 66 samples, 209 probes and 103 genes. If we want to analyze only a subset of samples, we could select samples using the "Step 1" shown above. This step is optional and default is to select all the samples. Next we select a sample grouping for the analysis. We could choose to cluster on genes, samples, both genes and samples or none using the combo box shown above. Check the box "Compute LR" to compute predictive ability pvalue. Let's select "CellType" sample grouping, leave step 1 as it is to select all the samples, cluster on "none" and click Analyze button.

If we had checked the compute LR box we would see an additional column "Gene list Statistics" with the computed pvalue in the results tables as shown in figure8.

Figure 8depicts a typical summary table details of which can be found in

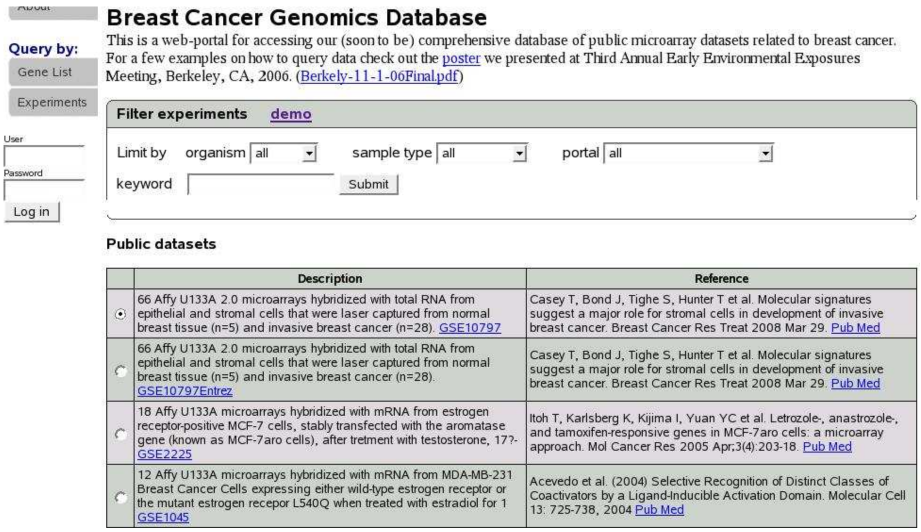

Figure 5: show experiments

Section Interpreting Results'.

2.2 Search for genes of interest using entrez id, symbol or description.

This section describes how to search for genes in the database and proceed with the analysis of genes found in search results.

Figure9 shows gene search page. This can be retrieved by clicking on the "Gene List" tab in the left menu. Genes could be searched by one of the three parameters: Gene ID (Entrez ID) e.g. 2099, symbol e.g. "ESR" or description e.g "estrogen". Type the value in the text box shown above. We can limit the search to a specific organism if required e.g. human, mouse or rat. For this example let's search for symbol "ESR". To do this, first select "Symbol" radio in the left column, type "ESR" in the text box. Select human from the Organism combo box (default is to look across all organisms) and click submit.

Figure10 shows the search result. Now we have all the genes with symbol "ESR" or similar symbol names. To analyze gene(s) select all the genes of interest and then click submit. Now we are presented a screen to select an experiment to analyze. From this point on, we proceed as explained in previous section "Using predefined gene lists".

2.3 Paste a list of genes in the box provided.

This section describes how to submit your own list of genes for analysis.

Figure11 shows the screen to submit your own list of genes. You can use either entrez ids (e.g. 2099) or symbols (e.g. esr1). As shown in the figure,

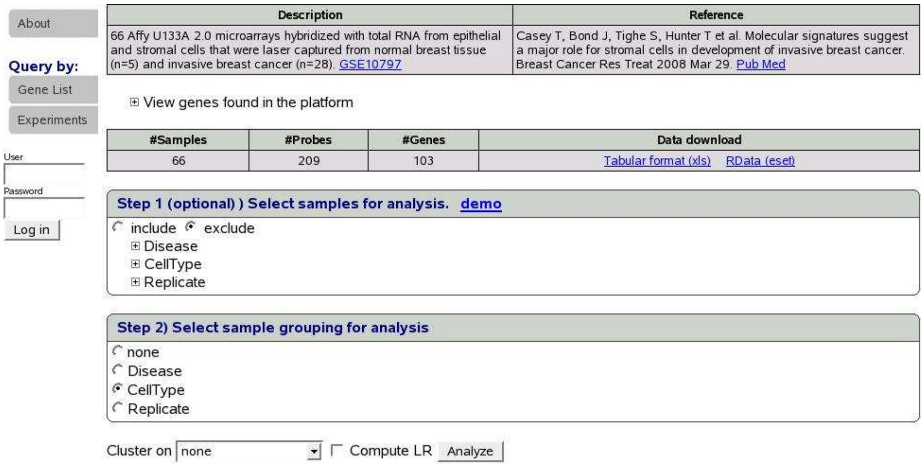

Figure 6: Get data

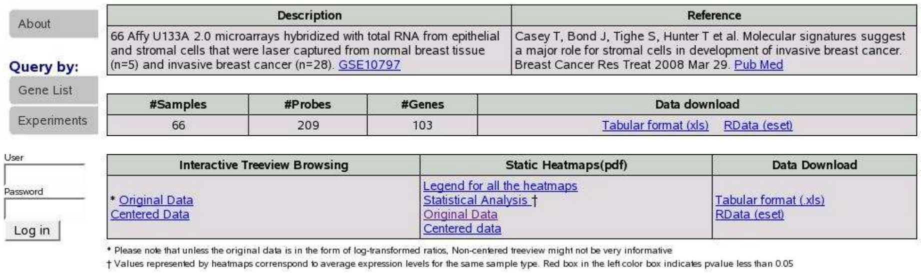

Figure 7: Results

type/paste a list in the box. We could optionally select an organism (human, mouse or rat) to filter these genes. By default, all the genes are submitted. Let's genes 10,12,2099 in the box and click submit.

At this point our database is searched for all the genes submitted and figure12 shows the list of genes found.

Now we can proceed as explained in the previous section 2.2"Search for genes of interest using entrez id, symbol or description".

2.4 Find predefined gene list(s) for your choice of genes.

This section describes how to find predefined gene lists that contain genes of interest.

There are two links beside title "demo" and "demo gene list". Clicking on demo gene list link shows a few sample genes that we are going to use for the purpose of this demo. The demo genes are as follows:

79575

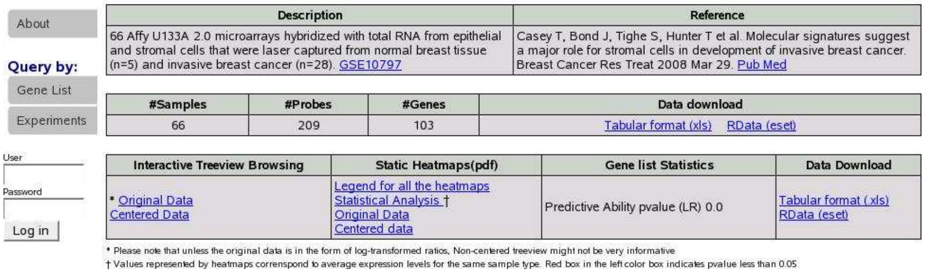

Figure 8: Results with LR

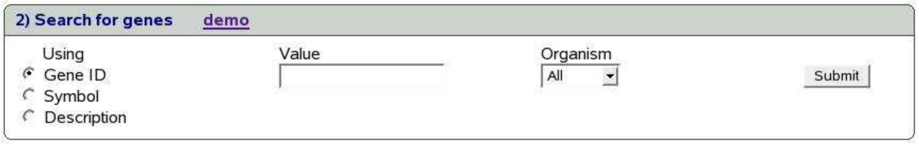

Figure 9: Search genes

10096
9447
Now copy and paste these genes in the text box above. The radio buttons provide option of how we want to search for the gene lists in the database. "Match any gene" would find all the gene lists that contain any of the genes we input whereas "Match all genes" would find only the gene lists that contain all of the genes.

We also have option of selecting which categories of predefined gene lists to search.

Figure14 shows all the categories of predefined gene lists that visible after clicking "Search in following lists".

By default all the lists are selected.

Let's proceed with our example using "Match all genes" option and default case for searching lists (search all lists).

Figure15 shows the result of our query. It shows all the gene lists found along with their description.

Let's select first list (NFkB) and submit.

Figure16 shows the resultant screen which shows the summary of gene lists selected. You might recall that this is similar to the screen shown in first section 2.1"Using predefined gene lists" and rest of the analysis is as described in that section.

2.5 Find genes with a phrase in their RIFs.

This section describes how to find genes based on their RIFs.

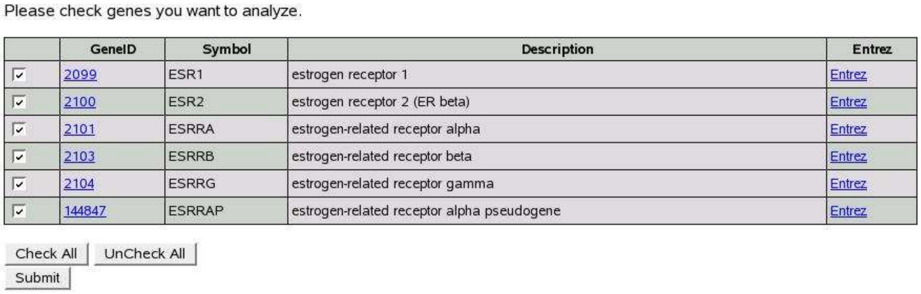
Please check genes you want to analyze.

	GeneID	Symbol	Description	Entrez
☑	2099	ESR1	estrogen receptor 1	Entrez
☑	2100	ESR2	estrogen receptor 2 (ER beta)	Entrez
☑	2101	ESRRA	estrogen-related receptor alpha	Entrez
☑	2103	ESRRB	estrogen-related receptor beta	Entrez
☑	2104	ESRRG	estrogen-related receptor gamma	Entrez
☑	144847	ESRRAP	estrogen-related receptor alpha pseudogene	Entrez

Figure 10: Gene search result

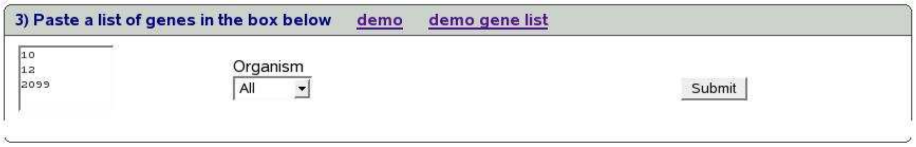

Figure 11: Submit a custom list of genes

The figure17 is self-explanatory. Let's type "argyrophilic grain disease" in the box and click submit.

Figure18 shows the result of our search. Select genes of interest and submit. Now we are presented a screen to select an experiment for analysis. Proceed as explained in previous section 2.2"Search for genes of interest using entrez id, symbol or description".

2.6 Find biogrid gene pairs for your gene(s).

This section describes how to find biogrid gene pairs for your genes.

Let's use genes 79575,10096,9447 we used in previous examples. Select "Match any genes" option and click submit.

Figure20 shows result for our search. Click submit and proceed with analysis as explained in section 2.2"Search for genes of interest using entrez id, symbol or description".

3 Start by selecting an experiment

If one is interested in a particular experiment, it is useful to locate the experiment first and then proceed with the analysis.

This section describes how to do this. At the time of this writing, there are 1904 experiments in the database and this number is continuously growing. Experiments are organized into different portals. If we know the portal the experiment of our interest belongs to, we can click on the portal name to list all

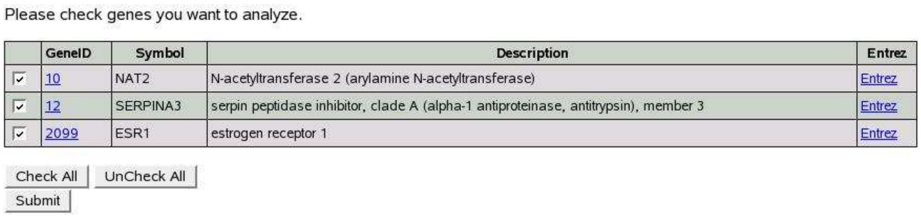

Figure 12: Submitted genes

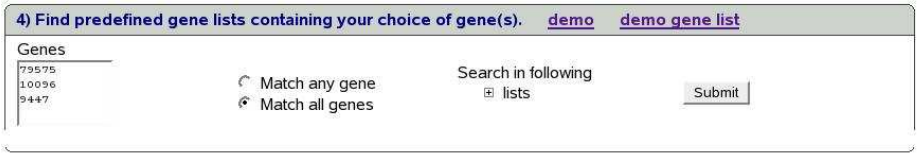

Figure 13: Find predefined gene lists

the experiments in that portal. Since this is not the case most times, a search function is provided to look for experiments of our interest.

Start by clicking "Experiments" tab on the left menu.

Figure 21 shows the experiment tab. The "Filter experiments" box on top provides search functionality.

The table below shows various portals and their descriptions. Clicking on a portal name shows the experiments belonging to that portal.

3.1 Search for an experiment

Search is a very important part of this portal given the number of experiments we have. To locate experiments of interest, a simple and effective search functionality is provided.

Figure22 shows the screen to filter experiments.

Following are the components of this module:

1) Organism: Experiments could be filtered by selecting one of the organism from the combo box named "organism". Three options proved are human, mouse and rat.

2) Sample type: Three sample types are provided for selection. Tissue, cell line and motif score. Select appropriate from the combo box.

3) Data type: Six data types are available for selection from the combo box.

4) Portal: All the available portals are listed here. Select a portal if you want to limit your search to that particular portal.

5) Keyword: This could be a name of experiment, a word in description or reference.

Let's search for experiments with keyword "miller" across all organisms, sample types and portals as an example.

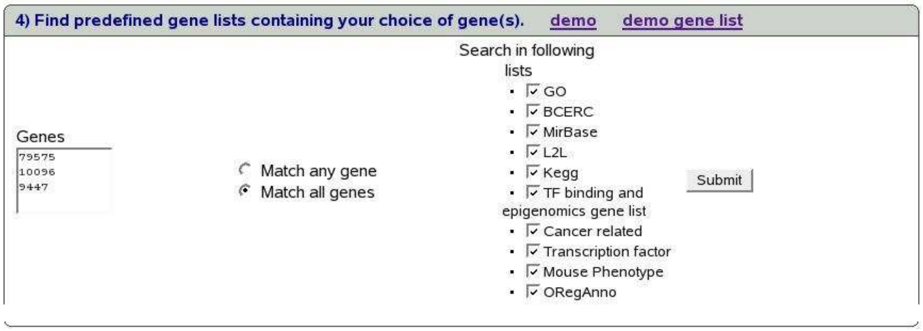

Figure 14: Select categories of predefined gene lists to search

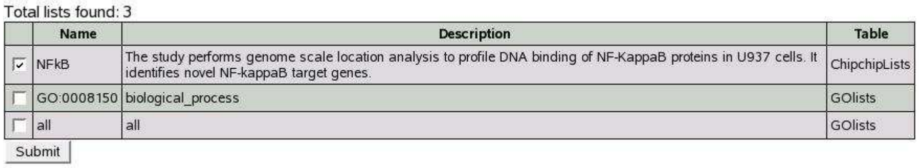

Total lists found: 3

	Name	Description	Table
☑	NFkB	The study performs genome scale location analysis to profile DNA binding of NF-KappaB proteins in U937 cells. It identifies novel NF-kappaB target genes.	ChipchipLists
☐	GO:0008150	biological_process	GOlists
☐	all	all	GOlists

Submit

Figure 15: Result for finding predefined gene lists

Figure 23 shows a part of the result page. As shown above, all the experiments found for the search criteria are listed in a table. Notice the "Query" and "Cluster" buttons in the last two columns of the table. These buttons provide a way to analyze the experiments and are explained in detail in following sections.

3.2 Query experiment

Clicking "Query" button shown in figure 23 opens a new window for that particula experiment.

e.g. Figure 24 shows the query page for experiment "GSE1045" in portal Breastcancer. The top table provides a summary of the experiment and bottom table lists all the properties (sample subgroupings) available for this experiment. These are useful for analysis.

These two tables are followed by all the options to construct a gene list shown in "Gene List" tab.

The procedure for analysis is similar to what was described in section 2 "Start by constructing gene list" except that the step to select experiment is skipped as we already have an experiment to work with.

3.3 Cluster experiment

Clicking "Cluster" button shown in figure 23 opens a new window for that particular experiment.

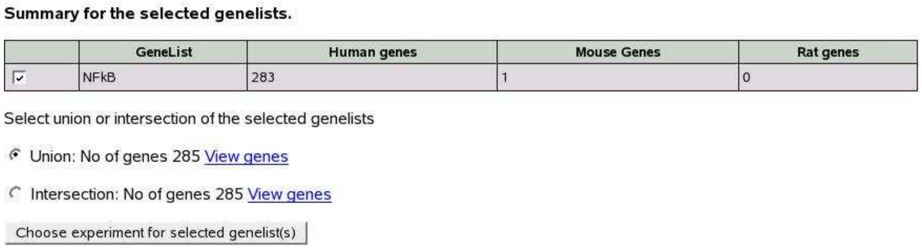

Figure 16: Summary of gene list

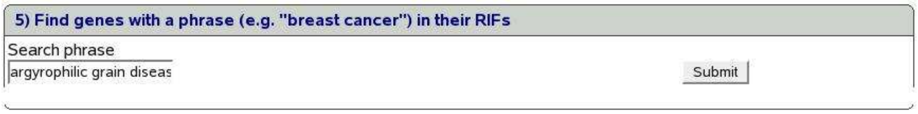

Figure 17: Find genes from their RIFs

4 Miscellaneous modules

4.1 Filter samples and select sample grouping for analysis

Note: This step is optional and default is to select all the available samples for analysis.

An experiment may have a number of samples which are organized in different groups (sample subgroupings).

One may wish to restrict analysis only to a subset of all the available samples for an experiment.

This section describes how this is achieved.

Figure 26 shows the screen to filter samples for experiment GSE10797. We can choose to either include or exclude all the samples that satisfy the criteria we are going to define by selecting appropriate option using the radio button.

All the sample subgroupings are listed in this box. When we click on a sample subgrouping, the link expands to show all the unique values for the same as shown in figure 27.

Let's say, we want to include only the samples for which Disease is cancer and CellType is epithelial.

As shown in figure 28, select include from the radio button, and check cancer box under Disease and epithelial box under CellType. When you click Analyze, only the samples for this criteria will be used for analysis.

Next step is to select sample grouping for analysis.

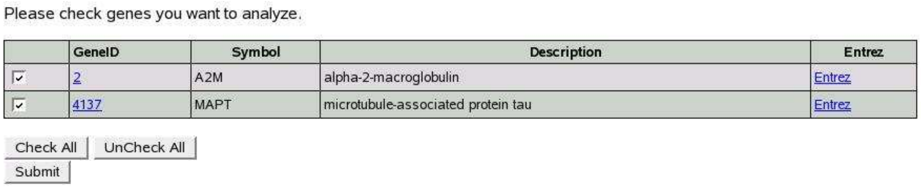

Figure 18: RIF search result

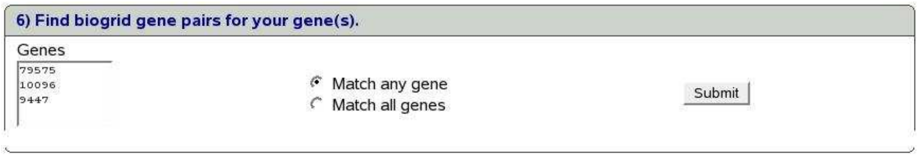

Figure 19: BioGrid

5 Interpreting Results

This section will describe the results page in detail. For illustration purpose we will take all genes with "stem cell" keyword in GO category as shown in figure 30.

Check all the genelists on the resultant page and proceed as explained in section 'Using predefined gene list(s)'. Select experiment "GSE2225" in portal "Breast cancer" and use sample subgrouping "Treatment" and cluster on "genes" to obtain the results shown in figure 31.

The results page structure is as follows. The first table gives a brief description of the selected experiment. The second table summarizes the data retrieved for the analysis of submitted query gene list and provides links for download. Data can be retrieved in the form of spreadsheet and R data object. The third table gives the analysis results which are explained in detail as follows.

5.1 Interactive Treeview Browsing

Unsupervised clustering of the query data was performed using the Bayesian model-based procedures [1] as well as simple hierarchical clustering. The functional annotation of the clustering structures was performed using the CLEAN framework [2], the integrative browsing of the data and functional annotations is facilitated through the Functional TreeView (FTreeView) which is a Java web-start based clustering browser [2]. Using FTreeView, one can identify clusters of genes based on their data profile and correlation with specific functional categories and use such gene lists to query and analyzed genomics data in other datasets.

We would like to note that in the case where no clustering option (on the genes as well as samples) is chosen, the TreeView application would show the heatmap with no dendrograms on either sides. This might make the heatmap

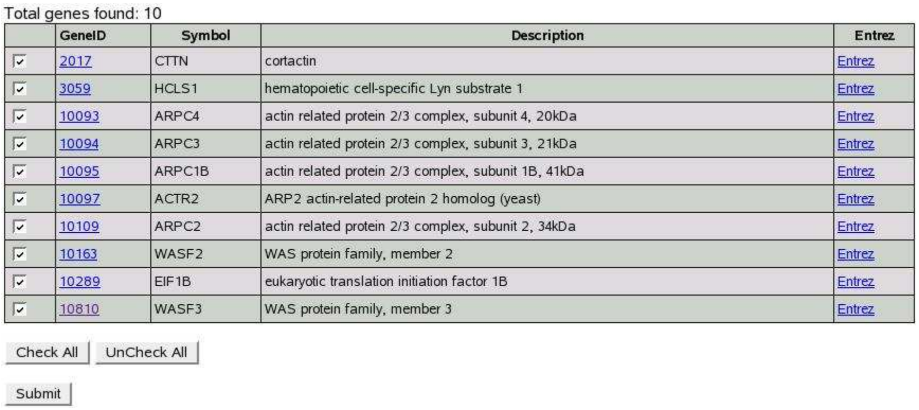
Total genes found: 10

	GeneID	Symbol	Description	Entrez
☑	2017	CTTN	cortactin	Entrez
☑	3059	HCLS1	hematopoietic cell-specific Lyn substrate 1	Entrez
☑	10093	ARPC4	actin related protein 2/3 complex, subunit 4, 20kDa	Entrez
☑	10094	ARPC3	actin related protein 2/3 complex, subunit 3, 21kDa	Entrez
☑	10095	ARPC1B	actin related protein 2/3 complex, subunit 1B, 41kDa	Entrez
☑	10097	ACTR2	ARP2 actin-related protein 2 homolog (yeast)	Entrez
☑	10109	ARPC2	actin related protein 2/3 complex, subunit 2, 34kDa	Entrez
☑	10163	WASF2	WAS protein family, member 2	Entrez
☑	10289	EIF1B	eukaryotic translation initiation factor 1B	Entrez
☑	10810	WASF3	WAS protein family, member 3	Entrez

Check All UnCheck All

Submit

Figure 20: BioGrid search result

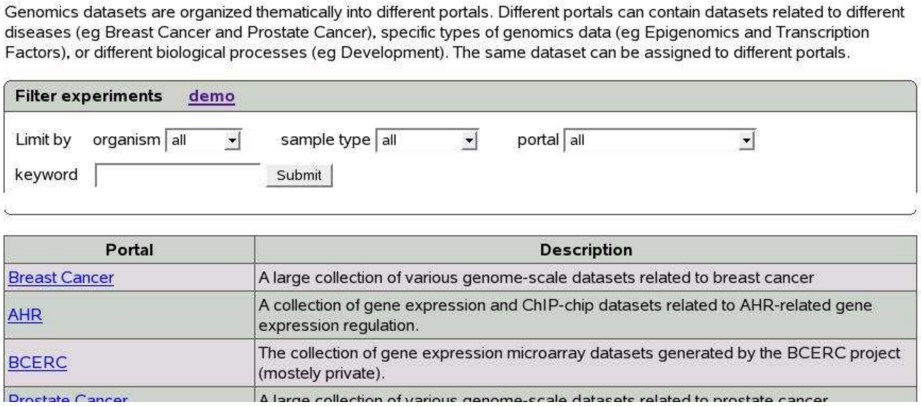
Genomics datasets are organized thematically into different portals. Different portals can contain datasets related to different diseases (eg Breast Cancer and Prostate Cancer), specific types of genomics data (eg Epigenomics and Transcription Factors), or different biological processes (eg Development). The same dataset can be assigned to different portals.

Filter experiments demo

Limit by organism all sample type all portal all

keyword Submit

Portal	Description
Breast Cancer	A large collection of various genome-scale datasets related to breast cancer
AHR	A collection of gene expression and ChIP-chip datasets related to AHR-related gene expression regulation.
BCERC	The collection of gene expression microarray datasets generated by the BCERC project (mostely private).
Prostate Cancer	A large collection of various genome-scale datasets related to prostate cancer

Figure 21: Experiments tab

incomprehensible at first. However, one can click on any of the genes or group of genes and the corresponding gene annotations will be displayed in the right-most window. The scenario is depicted in 33 where genes and samples are not clustered.

5.2 Static heatmaps

In addition to interactive treeview interface, Cluster analysis results are also available as static annotated heatmaps saved in pdf files. The values represented by heatmaps correspond to log transformed ratios.

Figure 34illustrates static heatmap clustered on selected stem cell genes across 6 treatment types. These sample annotations are provided separately in the link “legend for all the heatmaps” as shown in figure 35.

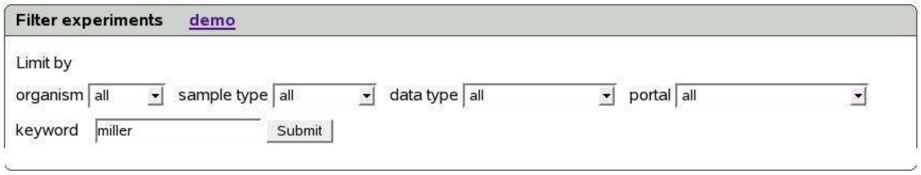

Figure 22: Search for experiments

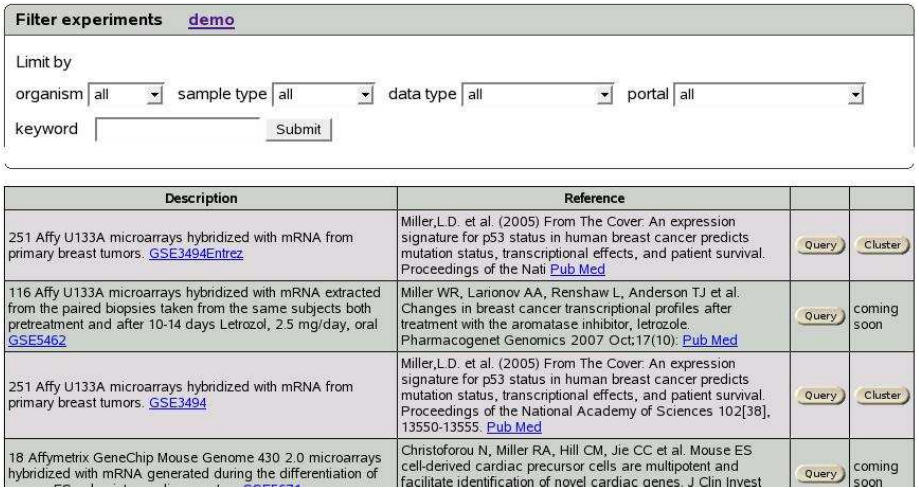

Figure 23: Filter experiment result

5.2.1 Statistical Analysis

For the selected samples in the dataset, we can identify differentially expressed significant genes. Values represented by heatmaps cornespond to average expression levels for the same sample subgrouping. Red box in the left sidebar indicates pvalue less than 0.05.

5.3 Gene List Statistics

5.3.1 Predictive Ability Pvalue (LR)

To assess the predictive ability of the selected sample grouping (in this exmample "treatment"), we select random genes of the same length as that of query gene list from the particular platform. The enrichment of the statistically significant genes in the query list was then assessed using logistic regression [3].

Description	Reference
12 Affy U133A microarrays hybridized with mRNA from MDA-MB-231 Breast Cancer Cells expressing either wild-type estrogen receptor or the mutant estrogen recepor L540Q when treated with estradiol for 1 GSE1045	Acevedo et al. (2004) Selective Recognition of Distinct Classes of Coactivators by a Ligand-Inducible Activation Domain. Molecular Cell 13: 725-738, 2004 Pub Med

Properties stored for the experiment:

genotype
estrodial
duration
sample

Figure 24: Query experiment

Description	Reference
18 Affy U133A microarrays hybridized with mRNA from estrogen receptor-positive MCF-7 cells, stably transfected with the aromatase gene (known as MCF-7aro cells), after tretment with testosterone, 17?-estradiol, two aromatase inhibitors (letrozole and anastrozole), an anti-estrogen (tamoxifen), and control. GSE2225	Itoh T, Karlsberg K, Kijima I, Yuan YC et al. Letrozole-, anastrozole-, and tamoxifen-responsive genes in MCF-7aro cells: a microarray approach. Mol Cancer Res 2005 Apr;3(4):203-18. PubMed

To view result of the cluster analysis for this dataset using the basic infinite mixture model (IMM) click on the button below. You need to have the Java Virtual Machine 1.42 installed on your computer to view the clustering. The results will open in a JavaTreeView viewer (Saldanha AJ. Bioinformatics 20: 3246-3248, 2004.). Cluster analysis was performed using gimmR package and the basic IMM model (Medvedovic M and Sivaganesan S. Bioinformatics 18: 1194-1206, 2002.). Genes were first pre-filtered based on variation and reliability of measurements in at least 10% of samples which resulted in 8,070 probes representing 6,160 unique genes. We selected best representative probes for each gene based on quality control considerations and clustered them using IMM. After clustering, we selected subclusters with average linkage posterior probability of clustering of at least 0.9. Samples were clustered using average linkage and the Pearson's correlation coefficient.

In order to run the applet below, you will need Java Runtime Environment 1.5 installed ().

Clustering Algorithm	No of Genes	Gene selection method	
DCGimm	5000	Copa	Cluster

Figure 25: Cluster experiment

5.4 Kegg Pathways for submitted genes

Query gene lists are incorporated into KEGG pathway images. One can click on a Pathway ID to view graphical representation of the pathway. Significantly expressed genes are painted yellow and other genes that were found in that particular pathway but are not significantly expressed are painted blue.

6 Case Study: Characterizing experimentally identified proliferation signature

We demonstrate the utility of the Genomics Portals through a case study investigating proliferation gene expression signature in rat mammary epithelium induced by different fatty acid diets [4]. This study established the increased proliferation of mammary epithelium as a consequence of several different dietary regiments in virgin female Spraque-Dawley rats. The study also identified a set of 85 genes whose expression levels were correlated with the increased proliferation.

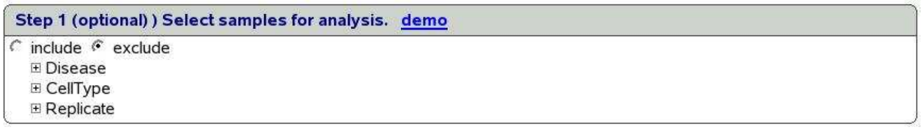

Figure 26: Select samples for analysis

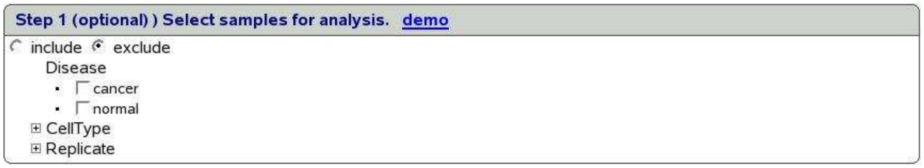

Figure 27: Sample selection expanded

6.1 Gene Expression data

We used Genomics Portals to study the functional importance of these 85 genes in five different biological processes examined in 4 gene expression datasets [5, 6, 7, 8] which are available in the portal. Here, we present step-by-step instructions for reproducing the results using Miller et.al. [5] dataset which comprises of 251 primary human breast tumors. This dataset was re-processed and curated before being deposited into the back-end databases under the id "GSE3494Entrez". The comparison of interest in this case was between the largest (top quartile) and smallest (bottom quartile) tumor with the assumption that large tumors are "more proliferative" than small tumors.

6.1.1 Select a dataset from the portal

Go to "Experiments" tab and type 'GSE3494Entrez' in the keyword field of 'Filter experiments' option. You can also find this experiment under 'Breast Cancer' portal. Press "submit". This will fetch the corresponding experiment and then press "Query" button.

6.1.2 Paste a query gene list

Paste a list of Entrez ids of 85 up regulated proliferation genes found at `http://eh3.uc.edu/documentation/upregulatedDietsGenes.txt` in the box (option 3) and press "submit".

6.1.3 Select Sample Grouping

This page provides collective information about the selected dataset, gene list submitted (and the actual number of probes found on this platform) as well as sample groupings associated with this dataset. In this example, select "tumorSize-quartiles" as sample grouping in step 2. We do no want to filter any samples

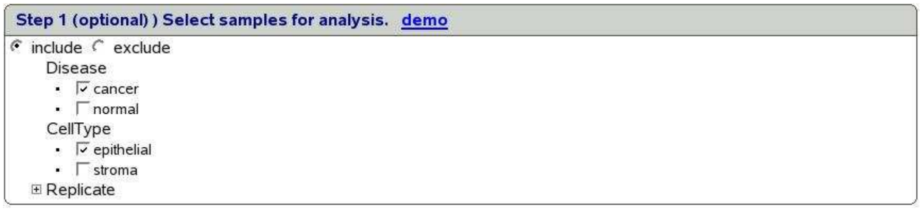

Figure 28: Filter sample example

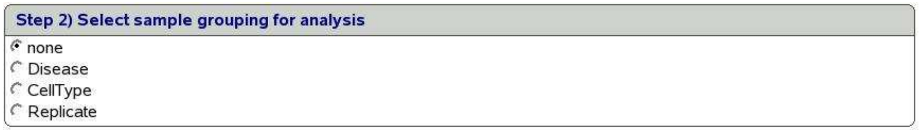

Figure 29: Select sample group

hence we can skip step 1. Also, select "computeLR" and press "Analyze" button. Figure 37depicts the snapshot of this step.

6.1.4 Results

Click on the "statistical Analysis" link and you will get a heatmap as shown in 39. The corresponding legends can be found by clicking on the link "legend for all the heatmaps" as shown in 38. One can see that indeed the genes in the query list are up-regulated in large tumors (quart-4) and are enriched for differentially expressed gene (LRpath p-value<10-9).

Similar analysis could be performed on the other 3 datasets using the same list of 85 up regulated proliferation genes. We have established the universality of the proliferation signature identified in the rat dietary studies across four very different biological systems using the Genomics Portals interface. The entire process of querying and generating results can be completed in less than 10 minutes. More details could be obtained from the manuscript.

6.2 ChIP-seq data for different transcription factors

In addition to using gene expression data, we further characterize our proliferation signature using ChIP-seq data for E2F1 transcription factor (TF) [9]. In the original paper, an extended set of genes identified through cluster analysis was linked to regulatory domain of E2F transcription factors by examining the overlap with E2F targets established in ChIP-chip [10] and global expression profiling [11] experiments, and computationally predicted E2F targets. Here, we used Genomics Portals to examine the newer ChIP-seq dataset assessing DNA binding of 15 different transcription factors, including E2F1, in mouse embryonic stem cells. Following steps can be conducted to obtain the respective heatmaps.

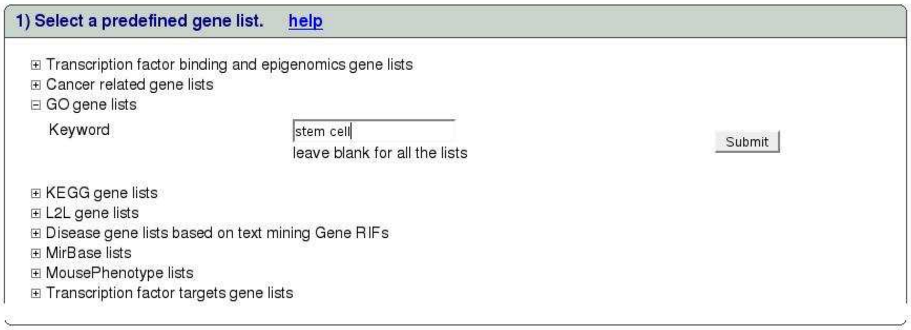

Figure 30: Select gene list with “stem cell” keyword

6.2.1 Select a data set from the portal

Go to “Experiments” tab and type ’GSE11431peaks’ in the keyword field of ’Filter experiments’ option. You can also find this experiment under ’Transcription Factors’ portal. Press “submit”. This will fetch the corresponding experiment and then press “Query” button.

6.2.2 Paste a query gene list

Paste a list of Entrez ids of 85 up regulated proliferation genes in the box (option 3) and press “submit”.

6.2.3 Select Sample Grouping

Select “Transcriptionfactor” as sample grouping in step 2. We do no want to filter any samples hence we can skip step 1 and then press “Analyze”

6.2.4 Results

Click on the link “Centered data” under static heatmap column of the result table. Figure 41shows heatmap of 15 Tfs and figure 40displays corresponding legends for each of the TFs.

We can see that in addition to most of the genes having a ChIP-seq peak for E2F1 within the regulatory region examined (-4kb to +1kb around TSS marked by 0), there were several other transcription factors such as N-myc,Tcfp2l1,c-Myc etc. that seem to have unusually many peaks for these gene. We can then focus on each of the TFs separately to take a closer look. We will illustrate the case using n-Myc TF.

We can select n-Myc TF out of 15 Tfs using “select sample” option in step 1 as shown in figure 42. Expand “Transcription Factor” and select n-Myc TF and click radio button “include” to select this sample. Then select “TranscriptionFactor” in step 2. select Cluster on “Genes” and “compute LR” options and click “Analyze”.

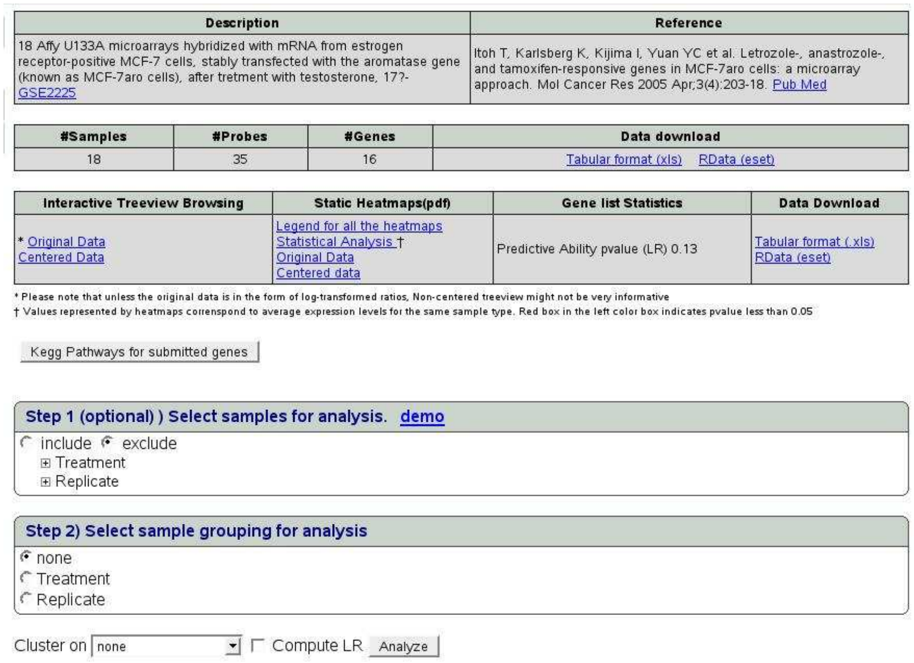

Figure 31: Results of stem cell gene list query

Then click on the link "Centered data" under static heatmap column of the result table. Figure 43 shows increased binding around TSS of the promoter region (-4kb to +1kb in this case) for some of these genes.

Here, we used the comparison to "random" sample by LRpath. Instead of the p-values, in this situation Genomics Portals by default uses the maximum "peak intensity" calculated for each gene across its whole regulatory region. Such statistical analysis confirmed that in addition to E2F1 (p-value $<$ 10-14), n-Myc (p-value $<$ 10-7), Tcfp2l1 (p-value $<$ .001), c-Myc (p-value $<$ .01), and Klf4 (p-value $<$ 0.01) all show signs of increased binding to regulatory regions of these genes.

6.3 Tri-methylation of histone across 5 human cell lines

We performed similar analysis on two epigenomics histone marks, H3k4me3 and H3k27me3 across five human cell line at different "differentiation" stages [12]. Following steps can be conducted to obtain the respective heatmaps.

6.3.1 Select a data set from the portal

Go to "Experiments" tab and type 'GSE11074' in the keyword field of 'Filter experiments' option. You can also find this experiment under 'Epigenomics'

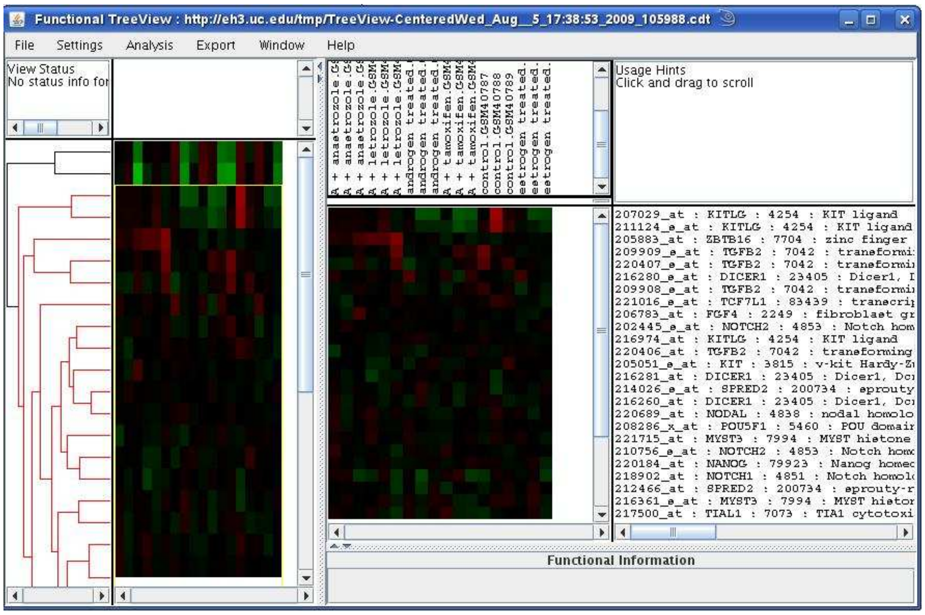

Figure 32: TreeView

portal. Press submit. This will fetch the corresponding experiment and then press “Query” button.

6.3.2 Paste a query gene list

Paste a list of Entrez ids of 85 up regulated proliferation genes in the box (option 3) and press “submit”.

6.3.3 Select Sample Grouping

In step1 (select samples for analysis), click on sample grouping name ’Histone’. This will show 2 options namely H3k4me3 and H3K27me3. We want to analyze the 2 histones separately. Choose H3K4me3 first by checking radio button ’include’. This step will filter samples in the analysis. In this case it will include only one type of selected histone. In step2, select “cell” as sample grouping for further analysis. Then choose clustering on “Genes” and press “Analyze”. Figure 44depicts the snapshot of this step.

6.3.4 Results

Click on “Centered data” link in the static heatmap column of the summary results table. Similar steps could be performed for other histone type. Figure 46 and figure 47show heatmaps of the 2 histones respectively. Figure 45shows legend for 5 cell types.

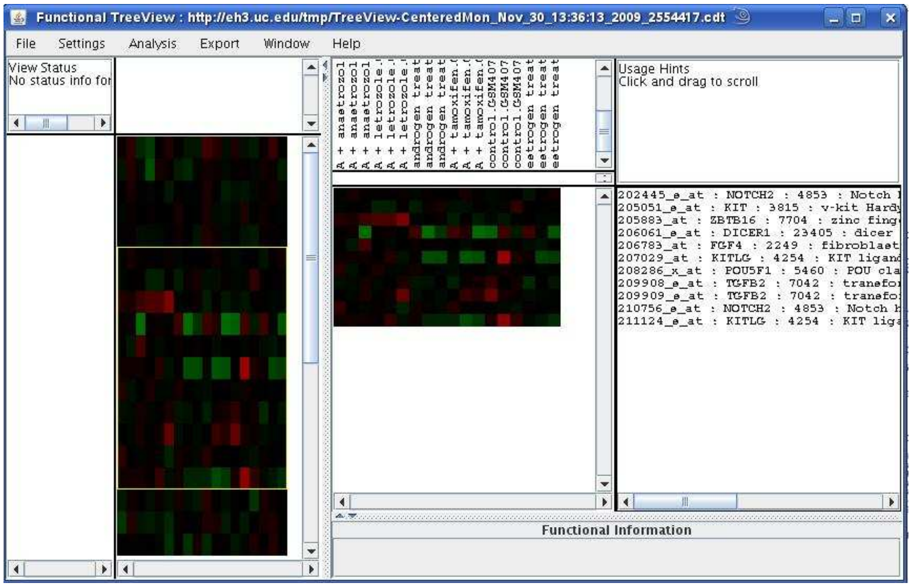

Figure 33: TreeView with no clustering of genes and samples

The results indicate that there is a subset of genes is in our proliferation signature with strong tri-mehylation of histone 3's lysine 4 across all 5 cell lines. On the other hand, tri-methylation of histone 3's lysine 27, in addition for differences between genes, also shows differences between different cell lines.

References

[1] Liu X, Sivaganesan S, Yeung KY, Guo J, Bumgarner RE, Medvedovic M: Context- specific infinite mixtures for clustering gene expression profiles across diverse microarray dataset. Bioinformatics 2006, 22:1737-1744.

[2] Freudenberg JM, Joshi VK, Hu Z, Medvedovic M: CLEAN: CLustering Enrichment ANalysis. BMC Bioinformatics 2009, 10:234.

[3] Sartor MA, Leikauf GD, Medvedovic M: LRpath: A logistic regression approach for identifying enriched biological groups in gene expression data 2. Bioinformatics 2008.

[4] Medvedovic M, Gear R, Freudenberg JM, Schneider J, Bornschein R, Yan M, Mistry MJ, Hendrix H, Karyala S, Halbleib D et al.: Influence of Fatty Acid Diets on Gene Expression in Rat Mammary Epithelial Cells. Physiol Genomics 2009.

[5] Miller LD, Smeds J, George J, Vega VB, Vergara L, Ploner A, Pawitan Y, Hall P, Klaar S, Liu ET et al.: From The Cover: An expression signature for

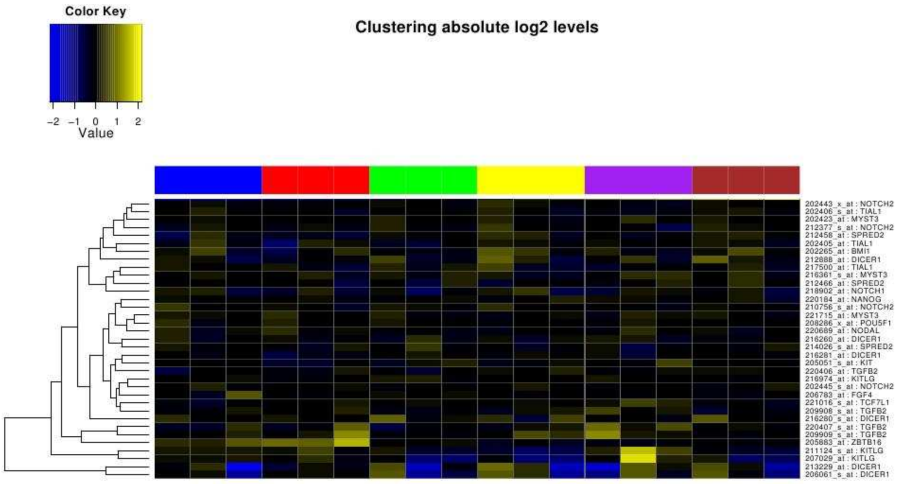

Figure 34: Static heatmap for stem cell genes

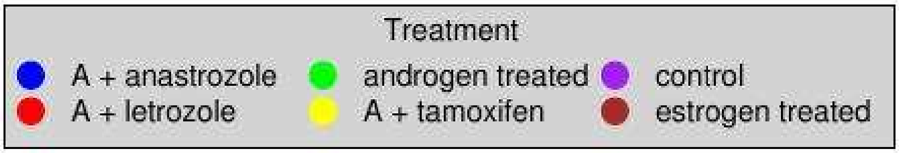

Figure 35: legend for 6 treatment types

p53 status in human breast cancer predicts mutation status, transcriptional effects, and patient survival. PNAS 2005, 102:13550-13555.

[6] Fournier MV, Martin KJ, Kenny PA, Xhaja K, Bosch I, Yaswen P, Bissell MJ: Gene Expression Signature in Organized and Growth-Arrested Mammary Acini Predicts Good Outcome in Breast Cancer. Cancer Res 2006, 66:7095-7102.

[7] Herschkowitz J, Simin K, Weigman V, Mikaelian I, Usary J, Hu Z, Rasmussen K, Jones L, Assefnia S, Chandrasekharan S et al.: Identification of conserved gene expression features between murine mammary carcinoma models and human breast tumors. Genome Biology 2007, 8:R76.

[8] Moggs JG, Murphy TC, Lim FL, Moore DJ, Stuckey R, Antrobus K, Kimber I, Orphanides G: Anti-proliferative effect of estrogen in breast cancer cells that re- express ER{alpha} is mediated by aberrant regulation of cell cycle genes. J Mol Endocrinol 2005, 34:535-551.

[9] Chen X, Xu H, Yuan P, Fang F, Huss M, Vega VB, Wong E, Orlov YL, Zhang W, Jiang J et al.: Integration of External Signaling Pathways with

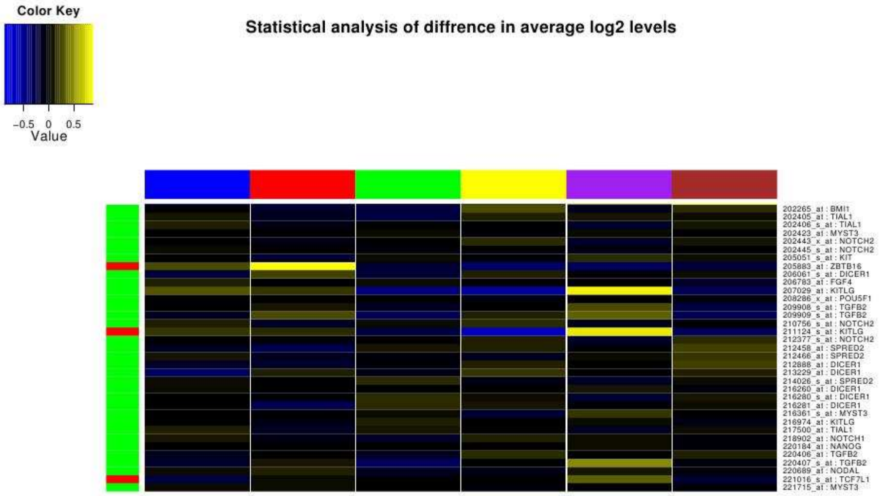

Figure 36: Statistical Analysis of stem cell genes

the Core Transcriptional Network in Embryonic Stem Cells. Cell 2008, 133:1106-1117.

[10] Xu X, Bieda M, Jin VX, Rabinovich A, Oberley MJ, Green R, Farnham PJ: A comprehensive ChIP-chip analysis of E2F1, E2F4, and E2F6 in normal and tumor cells reveals interchangeable roles of E2F family members. Genome Res 2007, 17:1550-1561.

[11] Kalma Y, Marash L, Lamed Y, Ginsberg D: Expression analysis using DNA microarrays demonstrates that E2F-1 up-regulates expression of DNA replication genes including replication protein A2 3. Oncogene 2001, 20:1379-1387.

[12] Mikkelsen TS, Hanna J, Zhang X, Ku M, Wernig M, Schorderet P, Bernstein BE, Jaenisch R, Lander ES, Meissner A: Dissecting direct reprogramming through integrative genomic analysis 2. Nature 2008, 454:49-55.

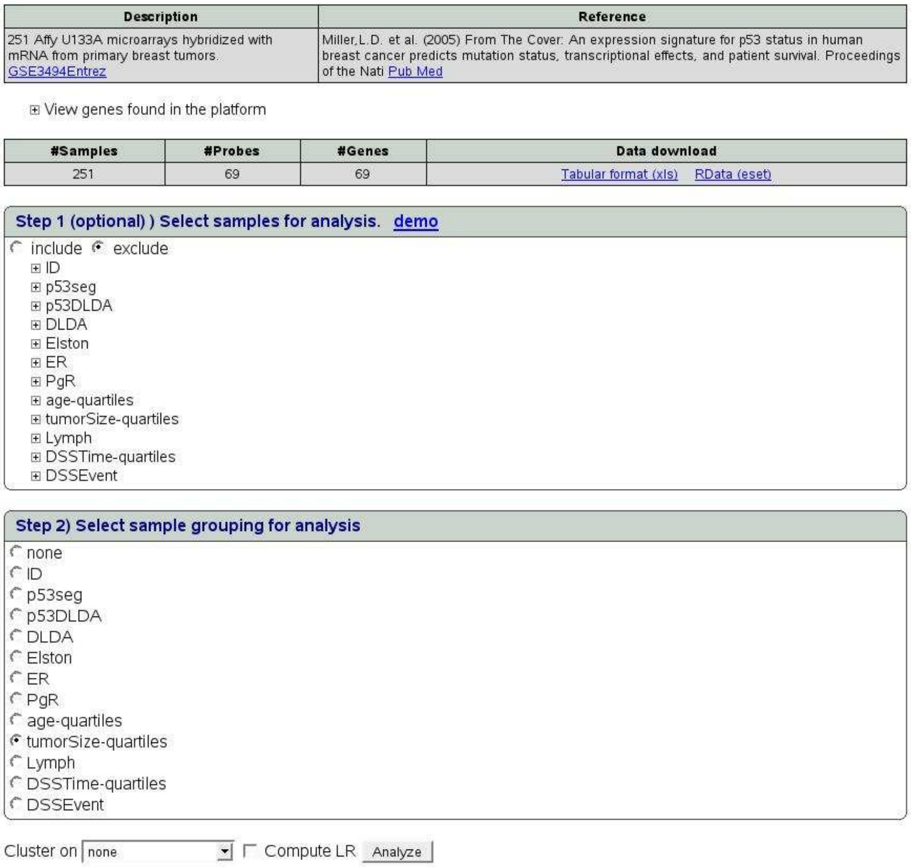

Figure 37: Proliferation genes on Miller dataset

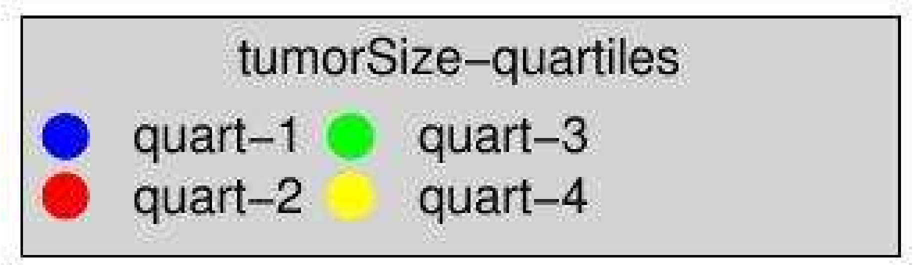

Figure 38: Legend for Tumor Size grade

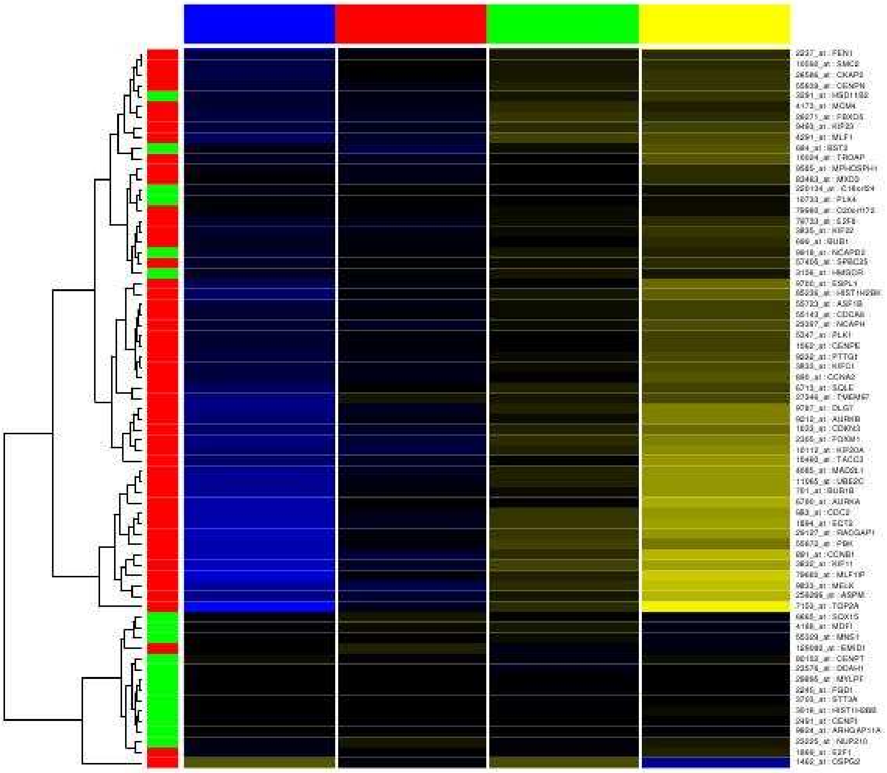

Figure 39: Statistical significance of up regulated genes

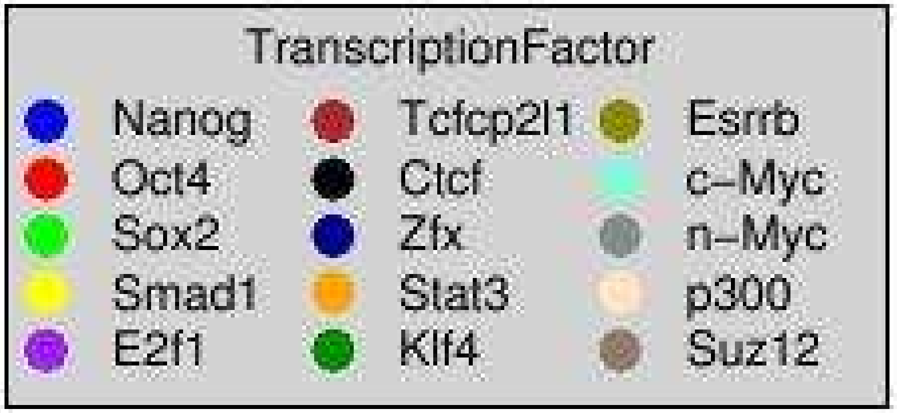

Figure 40: Legend for 15 TFs

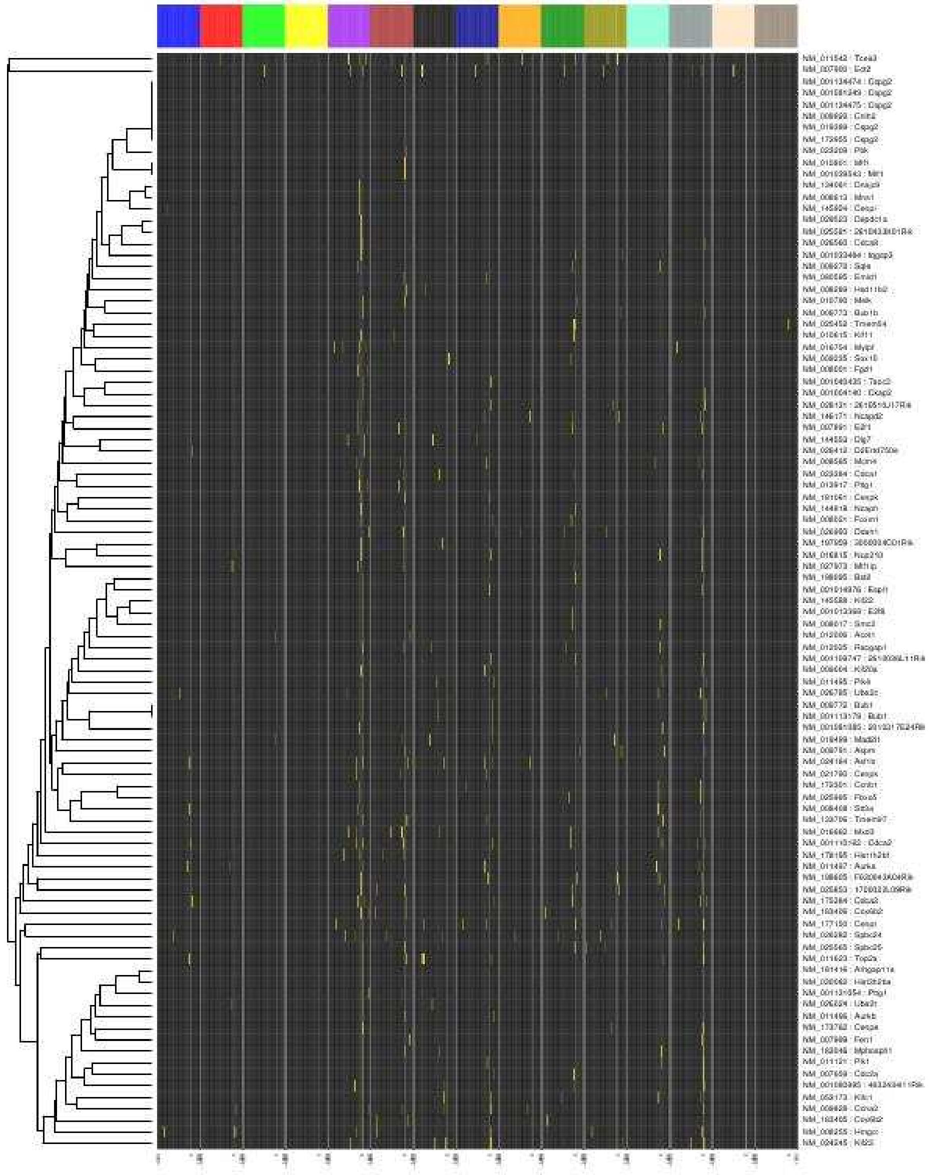

Figure 41: Heatmap of 15 TFs

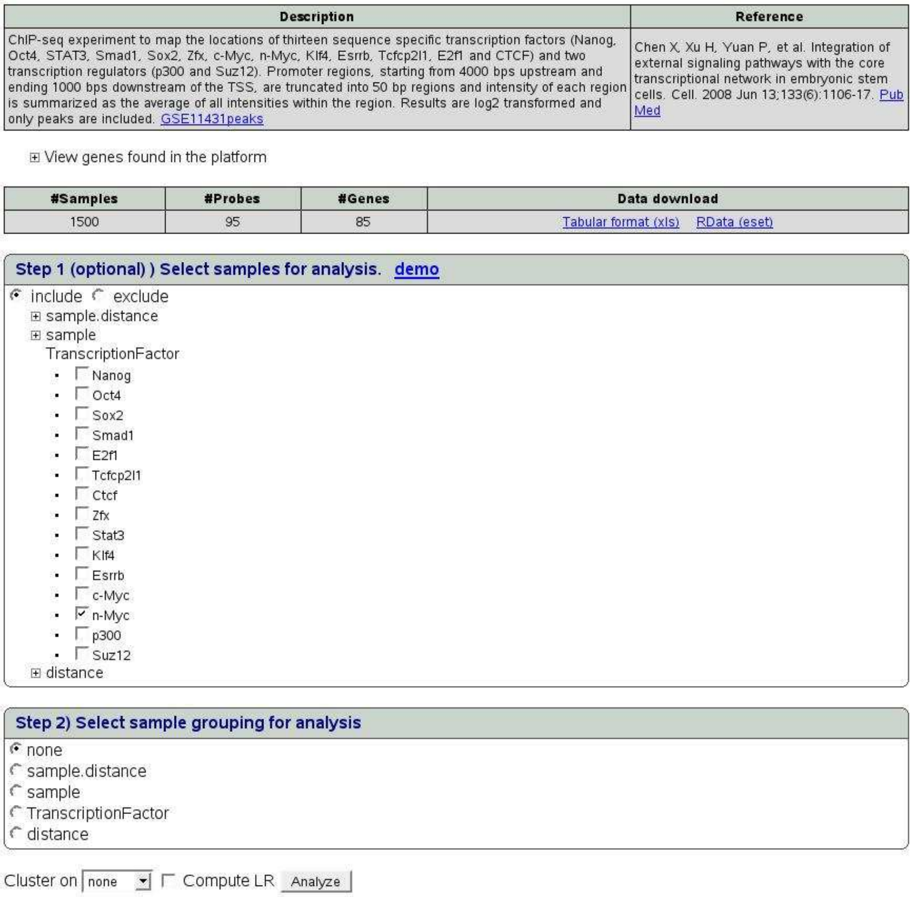

Figure 42: Select n-Myc TF

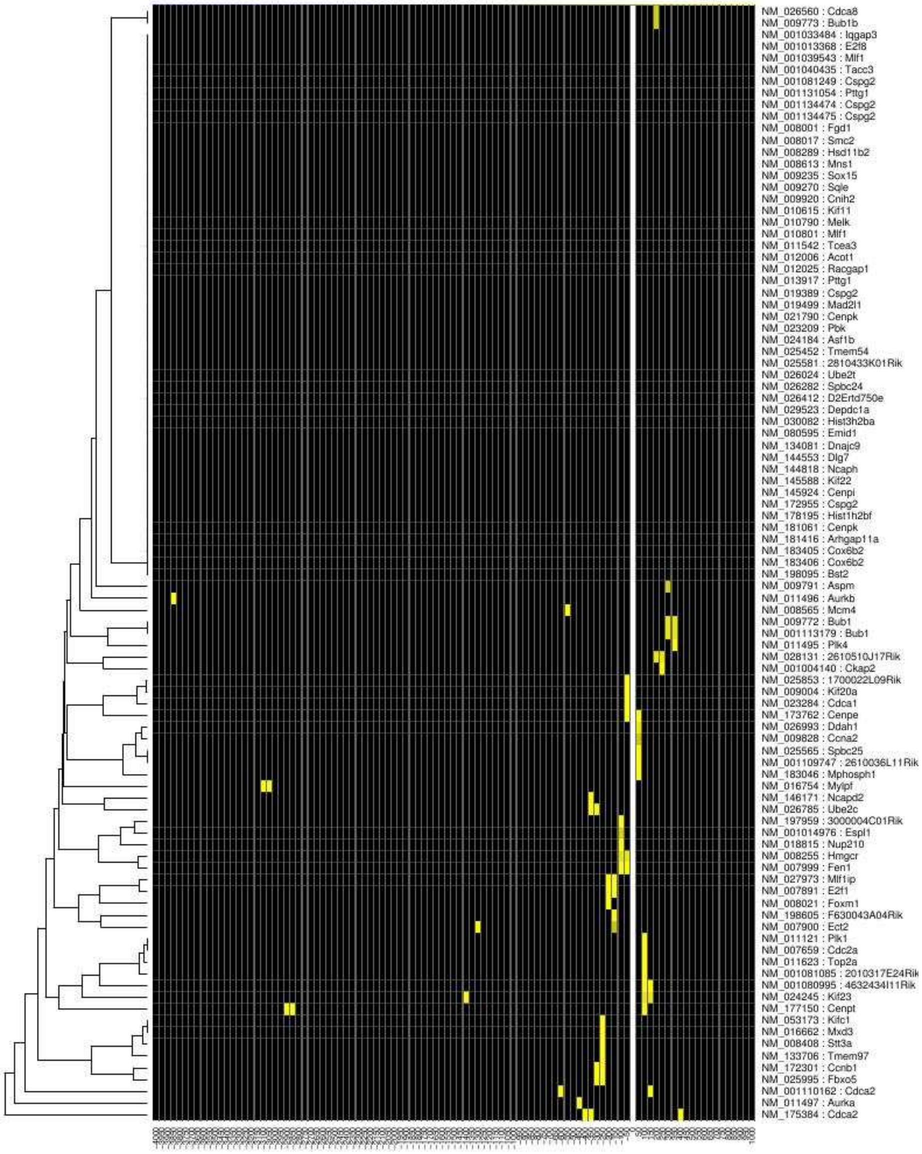

Figure 43: n-Myc TF heatmap

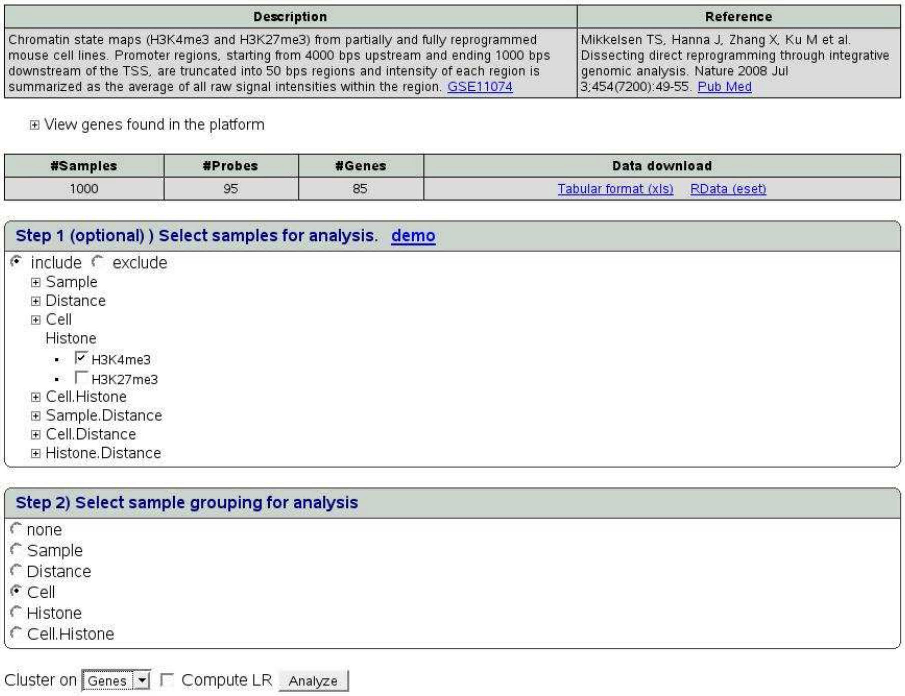

Figure 44: Filter H3k4me3 histone samples

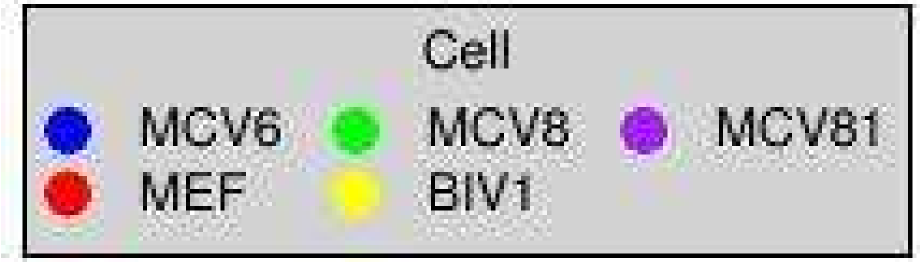

Figure 45: Legend for 5 cell types

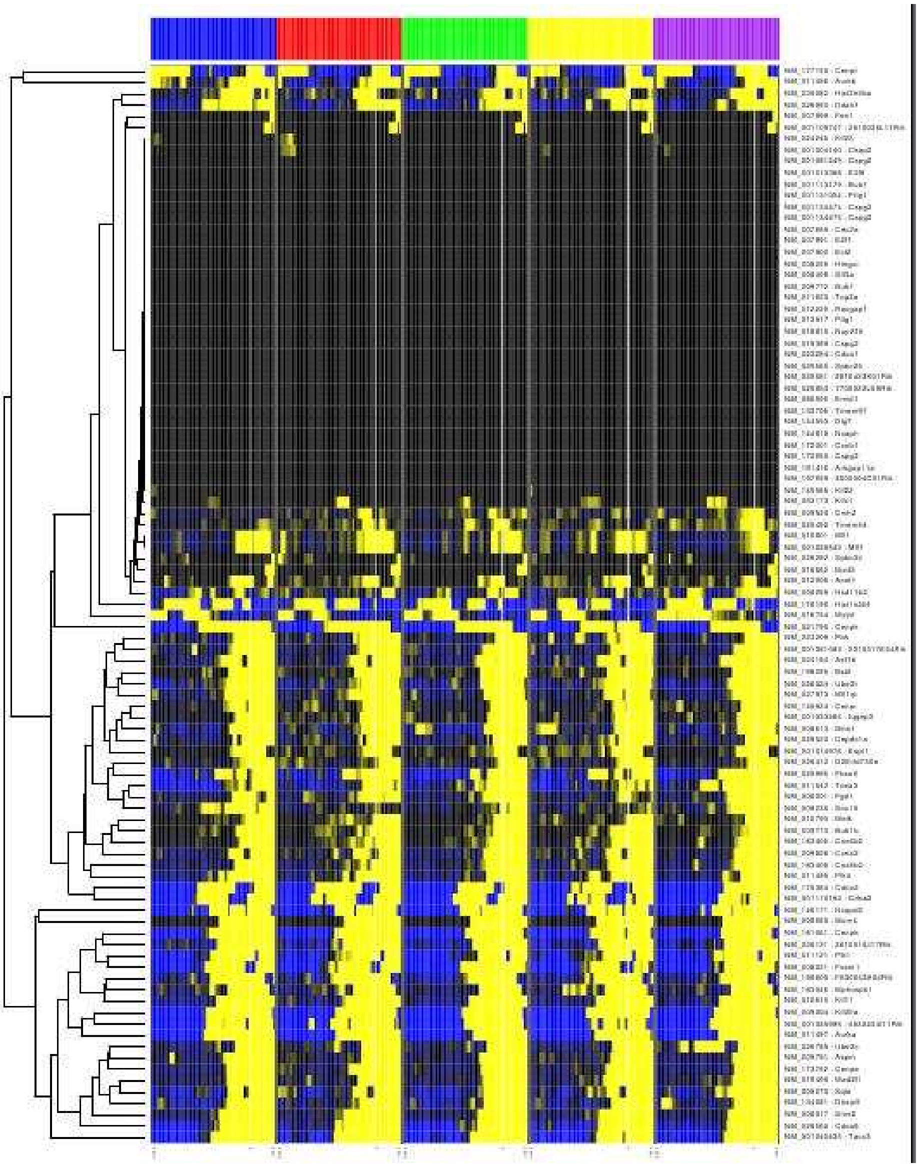

Figure 46: Histone H3k4me3 Heatmap

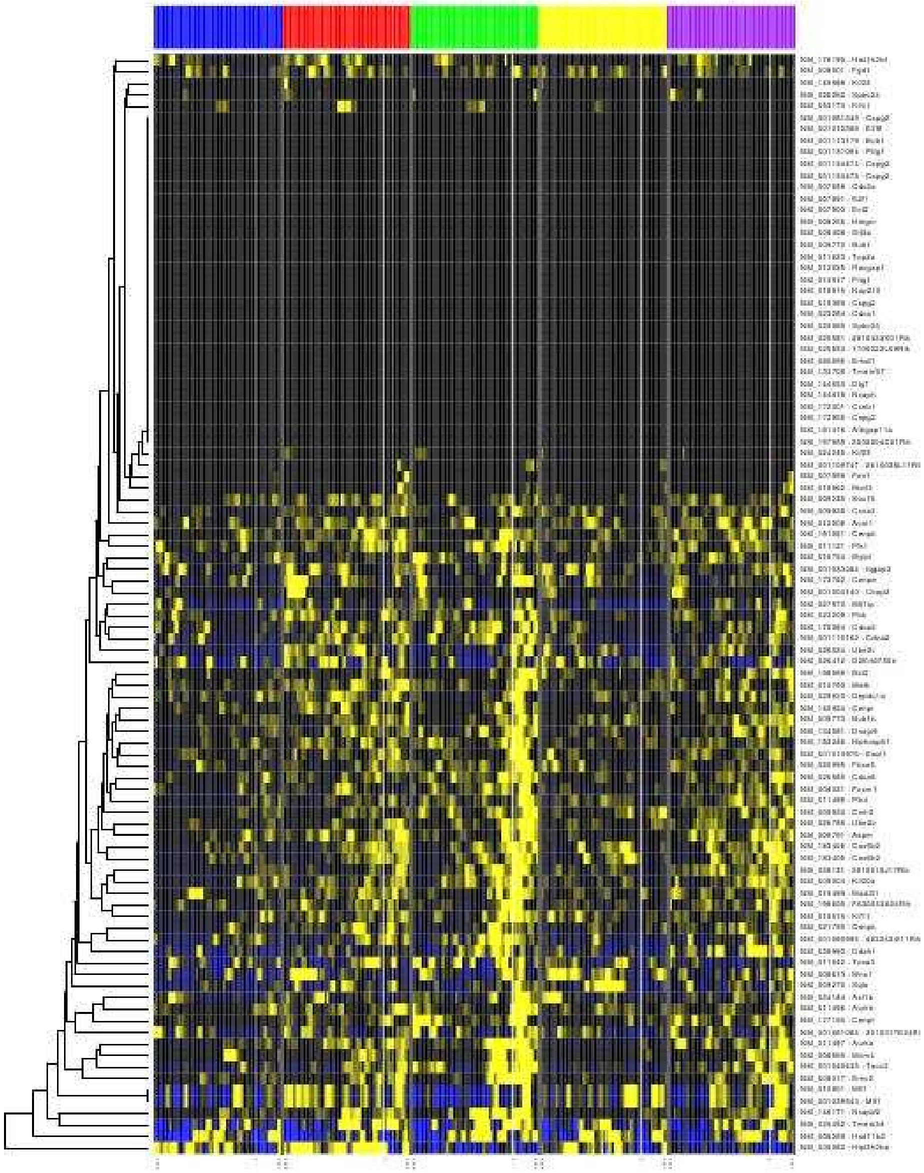

Figure 47: Histone H3k27me3 Heatmap
